# Chromatin Packing Domain Engineering Through the Manipulation of Nuclear Cationic States

**DOI:** 10.1002/advs.76786

**Published:** 2026-08-13

**Authors:** Cody L. Dunton, Carrillo Paola Gonzalez, Luay Almassalha, Wing Shun Li, Ruyi Gong, Nicolas Acosta, I Chae Ye, Jane Frederick, Christopher Hogg, Saira John, J. A. Gareth Williams, Rikkert J. Nap, Igal Szleifer, Vadim Backman

**Affiliations:** ^1^ Department of Biomedical Engineering Northwestern University Evanston Illinois USA; ^2^ Center for Physical Genomics and Engineering Northwestern University Evanston Illinois USA; ^3^ Chemistry of Life Processes Institute Northwestern University Evanston Illinois USA; ^4^ Department of Gastroenterology and Hepatology Northwestern Memorial Hospital Chicago Illinois USA; ^5^ Department of Chemistry Durham University Durham UK; ^6^ Department of Chemistry Northwestern University Evanston Illinois USA; ^7^ Robert H. Laurie Comprehensive Cancer Center Northwestern University Chicago Illinois USA

## Abstract

Cells must preserve and rewrite transcriptional memory to maintain identity and adapt to stress, yet the physical mechanisms governing this process remain unclear. Recent work implicates genome geometry in encoding transcriptional memory, with nanoscopic chromatin packing domains (PDs) serving as structural units that stabilize or reprogram transcriptional states. One powerful, yet underexplored regulator of this architecture is the nuclear ionic environment. Divalent cations can stabilize PDs through charge screening and phosphate bridging, suggesting a direct physicochemical mechanism linking ions to genome organization. In this study, we show that manipulating nuclear divalent cations is sufficient to reshape chromatin architecture, transcriptional output, and cellular resilience in living human cancer cells. Selective cation depletion (BAPTA‐AM, APDAP‐AM) produces smaller, less compact PDs and diminishes H3K9me3‐enriched heterochromatic cores, structural anchors of transcriptional memory. In contrast, magnesium enrichment enhances packing density and domain maturation. Live‐cell nanoscopy reveals that chromatin packing scaling responds within minutes to ionic perturbation. Transcriptomic profiling demonstrates coordinated gene expression changes and reduced adaptive plasticity, while chelator pretreatment increases chemotherapy sensitivity. Together, these findings identify nuclear ions as rapid, reversible regulators of chromatin packing domains and transcriptional memory, revealing ionic homeostasis as a fundamental mechanism linking genome architecture to cellular adaptation.

## Graphical abstract

Manipulation of intranuclear divalent cation concentrations rapidly and reversibly remodels chromatin packing domains in living cells. Magnesium depletion disrupts domain compaction, heterochromatin organization, and transcriptional regulation, whereas magnesium loading promotes more compact and stable domains. These structural transformations alter transcriptional plasticity and chemotherapeutic response, demonstrating that nuclear ionic conditions act as dynamic physicochemical regulators of genome architecture and cellular adaptation.

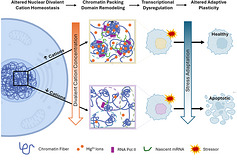

## 1 Introduction

Cells from all kingdoms of life possess a remarkable ability to maintain and reproduce specific transcriptional programs across cell divisions, allowing daughter cells to remember the transcriptional state of their parent cell, a phenomenon known as transcriptional memory [1, 2, 3]. This molecular memory enables cells to maintain identity, coordinate responses to stimuli, and adapt to environmental or physiological stressors. The loss of transcriptional memory contributes to vital cellular processes, such as differentiation [4], as well as pathologies such as aging [5], neurodegeneration [6], and cancer [7, 8, 9]. In these diseases, cells progressively “forget” their original transcriptional state and acquire aberrant transcriptional plasticity. Therefore, a central question remains: what is the mechanism by which transcriptional memory is encoded, stabilized, and rewritten within the nucleus?

While genetic and epigenetic mechanisms provide part of the answer, they cannot fully explain how transcriptional states persist or are reprogrammed. Emerging evidence suggests that transcriptional memory is physically embedded within the geometric organization of chromatin across multiple length scales, including nanoscopic chromatin domains observed in both imaging and genomic studies. Super‐resolution imaging and chromatin tracing approaches have revealed that chromatin is organized into heterogeneous, mesoscale domains composed of condensed nucleosome assemblies spanning ∼50–300 nm in size, often corresponding to active or regulatory regions [10, 11, 12, 13, 14, 15]. These structures have been described using various frameworks, including chromatin domains, nanodomains, and nucleosome clusters, reflecting a growing consensus that chromatin is neither uniformly open nor uniformly compact, but instead organized into spatially segregated, metastable units.

Building on this body of work, we define chromatin packing domains (PDs) as a quantitative and physically grounded description of chromatin organization, characterized by their mass‐fractal packing geometry in situ rather than contact frequency alone [8, 16, 17, 18, 19]. Unlike topologically associating domains (TADs), which are inferred by population‐averaged contact frequency maps, PDs are experimentally observed structures that can be remodeled independently of higher‐order connectivity features [16, 17]. Within a PD, chromatin follows a power‐law relationship between mass and radius (M∼r^D^), where the fractal dimension *D* quantifies how efficiently DNA fills space across nanoscopic length scales. This mass‐fractal architecture generates continuous gradients in chromatin density rather than homogenous aggregates, producing dense domain interiors that provide structural support and more accessible peripheral regions [14, 15] that optimize access for transcriptional machinery [16, 20] and help maintain regulatory states across cell divisions [21].

The formation and maturation of PDs are governed by three fundamental principles: (1) Forced returns, such as those generated by cohesion mediated loop‐extrusion or active transcription, nucleate nascent domains by locally compacting chromatin; (2) macromolecular crowding regulates chromatin density and accessibility, controlling how enzymes and transcriptional regulators explore and modify the chromatin landscape; and (3) transcriptional efficiency depends non‐monotonically on local chromatin density, where an optimal “goldilocks zone” balances accessibility and crowding‐enhanced reaction kinetic [22, 23, 24, 25, 26]. Within this ideal range, transcription is reinforced through feedback between polymerase activity and chromatin structure, enabling the stabilization of mature domains and the preservation of gene‐specific regulatory memory. These principles establish a dynamic nuclear landscape in which PDs act as stable yet reprogrammable units that encode transcriptional potential through their geometry. Perturbations to this balance can either compact chromatin beyond polymerase accessibility or relax it beyond structural integrity, leading to transcriptional dysregulation [16]. In aging, for instance, disruption of chromatin 3‐D organization weakens domain insulation and compromises the maintenance of transcriptional memory, producing aberrant gene expression, reduced regenerative capacity, and loss of cellular identity [6, 27, 28]. These observations raise an important question: what molecular and biophysical mechanisms stabilize chromatin packing domains and preserve their regulatory states across changing cellular environments?

Divalent cations such as Mg^2+^ and Ca^2+^ are ubiquitous within the nucleus and participate in many DNA and RNA metabolic processes. Mg^2+^ serves as an essential catalytic cofactor for RNA Polymerase II, ATP‐dependent chromatin remodelers, and epigenetic enzymes, while also neutralizing electrostatic repulsion along the DNA backbone to support nucleosome folding and higher‐order chromatin assembly [29, 30, 31, 32]. Ca^2+^, in contrast, primarily functions as a secondary messenger in signaling pathways that regulate transcription factor activity and cellular responses to environmental stimuli. Because these ions are fundamentally required for nuclear function, large‐scale ionic perturbations are expected to broadly influence transcriptional processes and chromatin organization. However, beyond these established biochemical and structural roles, it remains unclear how physiologically relevant fluctuations in nuclear ion concentrations may contribute to the geometric regulation and stabilization of chromatin packing domains associated with transcriptional memory.

Beyond their established biochemical roles in transcription and nuclear metabolism, divalent cations also exert fundamental electrostatic effects on chromatin organization that have long been appreciated from classical studies of chromosome swelling and decompaction following ionic perturbation and chelation [33, 34, 35, 36]. Although post‐translational histone modifications have been extensively studied as regulators of PD assembly, they cannot fully neutralize the dense negative charge carried by the DNA phosphate backbone [37]. To preserve the stability and function of chromatin packing domains composed of thousands to millions of base pairs, additional mechanisms must balance these electrostatic forces. Among the candidates, nuclear ions emerge as powerful yet underexplored regulators of chromatin architecture. Divalent cations, such as Mg^2^^+^ and Ca^2^^+^, not only serve as essential cofactors for polymerases and epigenetic enzymes but also directly modulate chromatin compaction through charge, ion condensation, and DNA‐phosphate‐Mg^2+^ bridging interactions [29, 31, 37, 38, 39, 40, 41, 42, 43, 44]. Given its millimolar intracellular concentrations, Mg^2+^ is expected to be the dominant regulator of chromatin compaction under physiological conditions [29], whereas Ca^2+^ contributes only under specific or experimentally elevated conditions [45]. Together, these mechanisms reduce electrostatic repulsion between DNA segments and promote higher‐order chromatin assembly, facilitating the formation and maturation of packing domains even in the absence of active transcription [16].

The nucleoplasm and nuclear periphery harbor a complex ionic milieu, including monovalent and divalent cations, polyamines, and transition metals, with Mg^2+^ as the dominant intracellular divalent species. These ions possess the potential to directly modulate the nanoscale chromatin structure through electrostatic interactions with the negatively charged phosphate backbone of DNA. Prior work has suggested a differential impact between monovalent and divalent ions on this process [37, 46, 47]. While monovalent ions have a muted impact on nucleosome assemblies, the charge neutralization of divalent ions, particularly Mg^2+^, appears robust at length scales from individual DNA strands up to mitotic chromosomes. Although this process is not yet fully understood, modeling and ex vivo experiments suggest that these are mediated by complementary processes of charge neutralization and phosphate bridging [30, 31, 32, 37, 38, 39, 40, 42, 46, 47, 48, 49, 50, 51]. While this work has provided insights into how ions contribute to phosphate charge neutralization and chromatin assembly, it remains to be demonstrated if ionic manipulation can mechanistically regulate crucial cellular behavior through the transformation of chromatin structure.

Here, we hypothesize that physiologically relevant nuclear ionic perturbations contribute to transcriptional memory by influencing the biophysical assembly and stability of chromatin packing domains. To test this, we employed a multidisciplinary approach integrating advanced imaging techniques, biochemical assays, and computational modeling. Our experimental design encompasses the manipulation of nuclear ion concentrations through pharmacological interventions coupled with nanoscopic and high‐resolution imaging to measure the transformation of nanoscopic chromatin domains in real‐time. From this approach, we demonstrate that intranuclear cations predictably reshape chromatin packing domains and, in turn, regulate gene transcription. We then examine the functional consequence of ion‐mediated packing domain manipulation by showing that domain destabilization from magnesium and calcium chelation limits the chemoevasive potential of cancer cells. In summary, our research represents a concerted effort to provide nanoscale insight into how nuclear ionic perturbations influence chromatin packing dynamics and associated transcriptional behavior in living human cancer cells.

## 2 Results

### 2.1 Nuclear Ion Concentration Can Be Regulated With High Specificity

Although numerous groups have investigated the role of ions in regulating chromatin structure and cellular function [33, 34, 41, 51, 52, 53], important challenges remain in establishing physiologically relevant ionic conditions and understanding how these changes in the ionic nuclear environment reshape interphase chromatin organization. Owing to the complexity of the nucleoplasm and chromatin, many studies have been performed on isolated nuclei, chromosomes, nucleosomes, or DNA molecules. For example, isolated mitotic chromosomes from chicken erythrocytes, chromatin segments, or reconstituted nucleosomes were exposed to varying concentrations of salts dissolved in buffer [35, 38, 39, 50, 54, 55]. Similarly, isolated nuclei were exposed to different concentrations of ions dissolved in the extranuclear environment, then stained prior to fixation and imaging [56]. In live cells, ionic manipulation using ion channel inhibitors or altering extracellular ion concentration has been employed, but these manipulations have secondary consequences, including the activation of histone methylation enzymes [29, 34, 57].

To overcome these limitations, we modulated ions through reciprocal modalities to facilitate both the removal and delivery of ions into the nucleoplasm. Specifically, we first treated cells with an established divalent cation chelator conjugated with an AM ester to allow for cellular uptake, BAPTA‐AM [58], and a novel compound that is more specifically targeted to Mg^2+^, APDAP‐AM [59]. In contrast to extracellular chelators, these compounds reduce the divalent cations directly within the cellular and nuclear environment. To further support that any alteration in chromatin structure is directly due to ion manipulation, we exposed HCT116 cells to elevated concentrations of Mg^2+^ via treatment with a MgCl_2_ solution. This treatment increases cellular Mg^2+^ ion content and has been associated with changes in constitutive and facultative heterochromatin content [57]. Given the substantially higher intracellular concentration of Mg^2+^ relative to Ca^2+^, the observed structural changes are expected to primarily reflect Mg^2+^ depletion.

**FIGURE 1:**
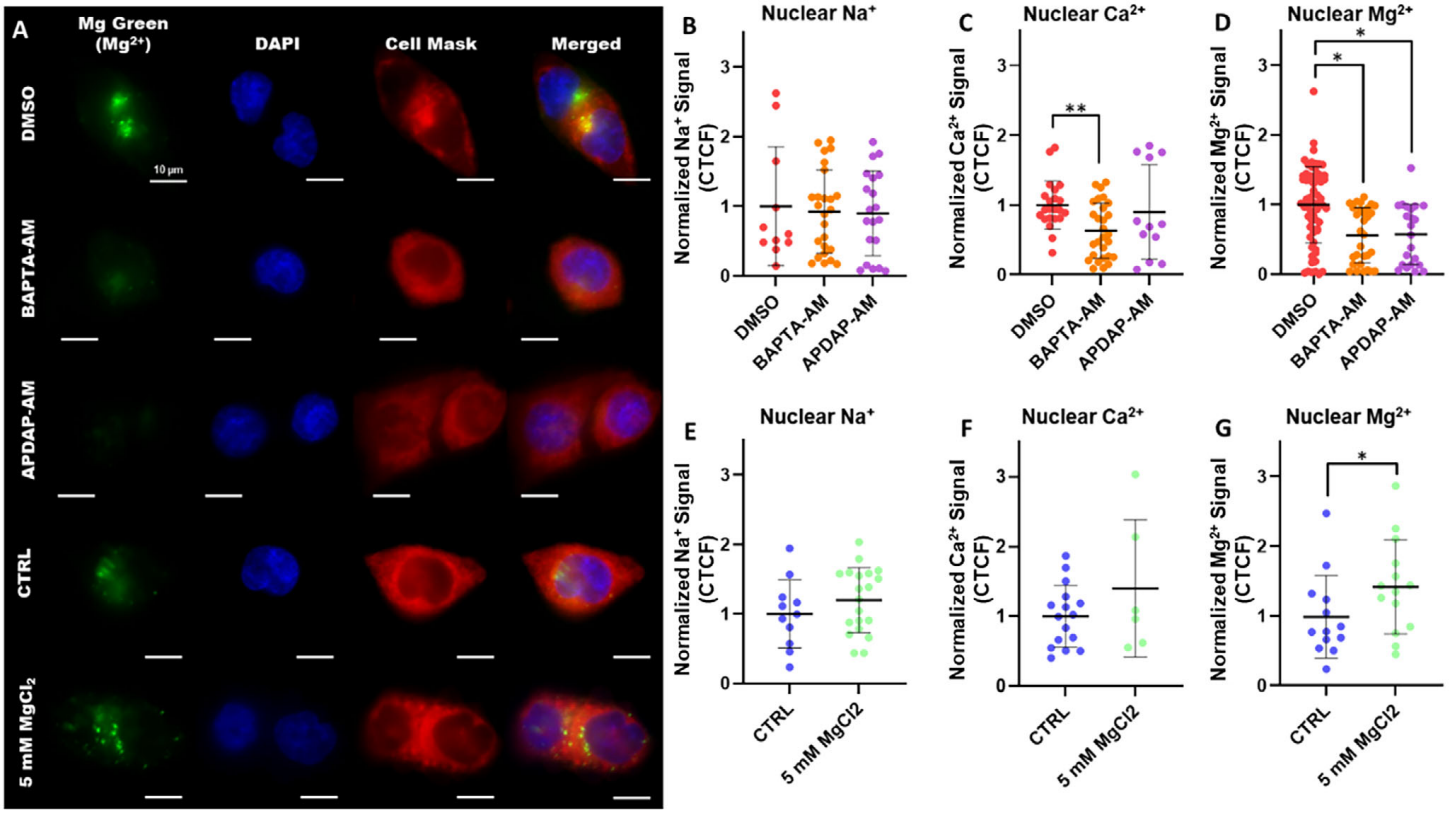
Manipulation of distinct nuclear ions via chelation and ion loading. (A) Representative images of HCT116 cells treated with ion chelators (BAPTA‐AM, APDAP‐AM) or exogenous Mg^2+^ (MgCl_2_) and stained for intracellular Mg^2+^ ion indicator (Magnesium Green AM), DNA (DAPI), and plasma membrane (CellMask). (B‐D) Quantification of corrected total cell fluorescence (CTCF) for nuclear Na^+^ (B), Ca^2+^ (C), and Mg^2+^ (D) in cells exposed to DMSO or divalent cation chelation for 60 min, normalized to the average CTCF of DMSO controls (n = 10 to 20 cells per condition labeled with Na^+^, n = 10 to 20 cells per condition labeled for Ca^2+^, n = 20 to 60 cells per condition labeled for Mg^2+^; N = 3 independent experiments). (E‐G) Quantification of CTCF for nuclear Na^+^ (E), Ca^2+^ (F), and Mg^2+^ (G) in cells exposed to 5 mM MgCl_2_ for 60 min, normalized to the average CTCF of untreated controls (n = 10 to 20 cells per condition for all ion labels; N = 3 independent experiments). Data are presented as the mean ± SD for each condition. Statistical significance was determined using one‐way ANOVA with Tukey's post‐hoc test (^*^ *P* < 0.05, ^**^ *P* < 0.01).

Intracellular ionic concentrations in HCT116 were thus modulated via intracellular chelation and loading. To quantify the relative change in concentrations, we utilized live‐cell fluorescent labeling of monovalent (Na^+^) and divalent (Ca^2+^, Mg^2+^) cations at short timescales (60 min, Figure 1A, Figure S2A,B). Although Mg^2+^ staining exhibited a spatially heterogeneous signal within the nucleus, quantification was performed over the entire nuclear region using corrected total cell fluorescence (CTCF), ensuring that measured differences reflect global changes in nuclear ion levels rather than localized intensity variations. Treatment with both BAPTA‐AM and APDAP‐AM for 60 min significantly and rapidly reduced nuclear ion staining of divalent cations with minimal impact on nuclear monovalent ion concentration (Figure 1B–D). Consistent with their reported affinities, BAPTA‐AM reduced both nuclear Mg^2+^ and nuclear Ca^2+^, while APDAP‐AM resulted in a significant depletion of nuclear Mg^2+^ with no significant change in Ca^2+^(Figure 1D,E). This selective depletion enables the distinction between Mg^2+^‐biased effects and broader divalent ion chelation. Having demonstrated the ability to deplete intranuclear divalent ions, we next investigated the change in intranuclear ion concentrations with cation loading. HCT116 cells were treated with 5 mM MgCl_2_, which increased the nuclear signal of magnesium ions after 60 min of treatment. Notably, cation loading was selective as Mg^2+^ did not significantly increase Na^+^ or vice versa (Figure 1E–G). Unexpectedly, cation loading produced larger variations in nuclear ion levels than targeted treatment with intracellular chelators within the cellular population. As such, this indicated that ionic concentrations within the nucleus were not solely governed by passive diffusion through the nuclear pore complex but appear to be a regulated process by active exchange transporters. Collectively, these tools demonstrate the ability to bi‐directionally, rapidly, and selectively regulate intranuclear ionic concentrations to study their role in chromatin structure in living cells.

### 2.2 Divalent Cations Regulate Interphase Nanoscopic Chromatin Geometry In Situ

Having demonstrated the ability to regulate intranuclear divalent ionic concentrations in interphase cells, we next tested whether depletion of intracellular divalent cations using BAPTA‐AM regulates the physical assembly of the genome in packing domains. Based on the framework of domain formation described previously [16], we hypothesized that divalent cations play a crucial role in initiating chromatin packing by providing charge screening between locally interacting chromatin elements. In this model, the early stages of domain formation rely heavily on ionic stabilization, while the maintenance of the mature domains becomes progressively less sensitive to ion fluctuations as additional structural reinforcements (i.e., histone methylation, enzymatic crosslinking) contribute to domain stability as the structure develops [16]. Consequently, we anticipated that ion depletion would differentially disrupt nascent or loosely assembled domains, while mature, heterochromatin‐rich structures would be relatively preserved. To investigate this, we utilized chromatin scanning transmission electron microscopy (ChromSTEM) tomography, which enables direct visualization and quantification of the polymeric structure of chromatin in situ at sub‐5 nm resolution [8]. ChromSTEM employs a photo‐activatable dye and diaminobenzidine (DAB) to proportionally label DNA for electron microscopy imaging [60]. High‐angle annular dark‐field tomography has previously demonstrated that chromatin organizes into disordered nucleosome fibers (<25 nm), heterogeneous packing domains (PD, 50–200 nm), and larger chromosomal territories (>200 nm) [8, 19]. While ChromSTEM has limited throughput and does not resolve individual loci, it uniquely captures supra‐nucleosomal length scales inaccessible with current super‐resolution imaging techniques [16, 17, 18].

Packing domains were quantified using chromatin volume concentration (CVC), radius (r), packing efficiency, and fractal dimension (D), which describes the mass‐radius scaling behavior of chromatin within a domain (M∼r^D^). Detailed definitions and derivations of these parameters are provided in the Methods. Briefly, CVC and radius reflect the density and size of the domains, while D quantifies how efficiently the chromatin polymer fills three‐dimensional space [10]. On average, packing domains span thousands to millions of base pairs corresponding to radii of ∼50‐150 nm (approximately 50 kbp‐5 Mbp). These domains exhibit broad distributions in sizes, densities, and scaling exponents, reflecting heterogeneous states of chromatin organization within the nucleus. This distribution potentially intersects with their function: small, low‐density structures likely represent loosely organized or transient domains; large, low‐density structures likely represent partially disassembled or structurally unstable domains; and high‐density domains likely represent efficiently packed (mature) structures. To quantify this heterogeneity at the nuclear level, we define D_n_ as the average fractal dimension across the nucleus, which accounts for both the number of domains and the fraction of nuclear volume they occupy.

**FIGURE 2:**
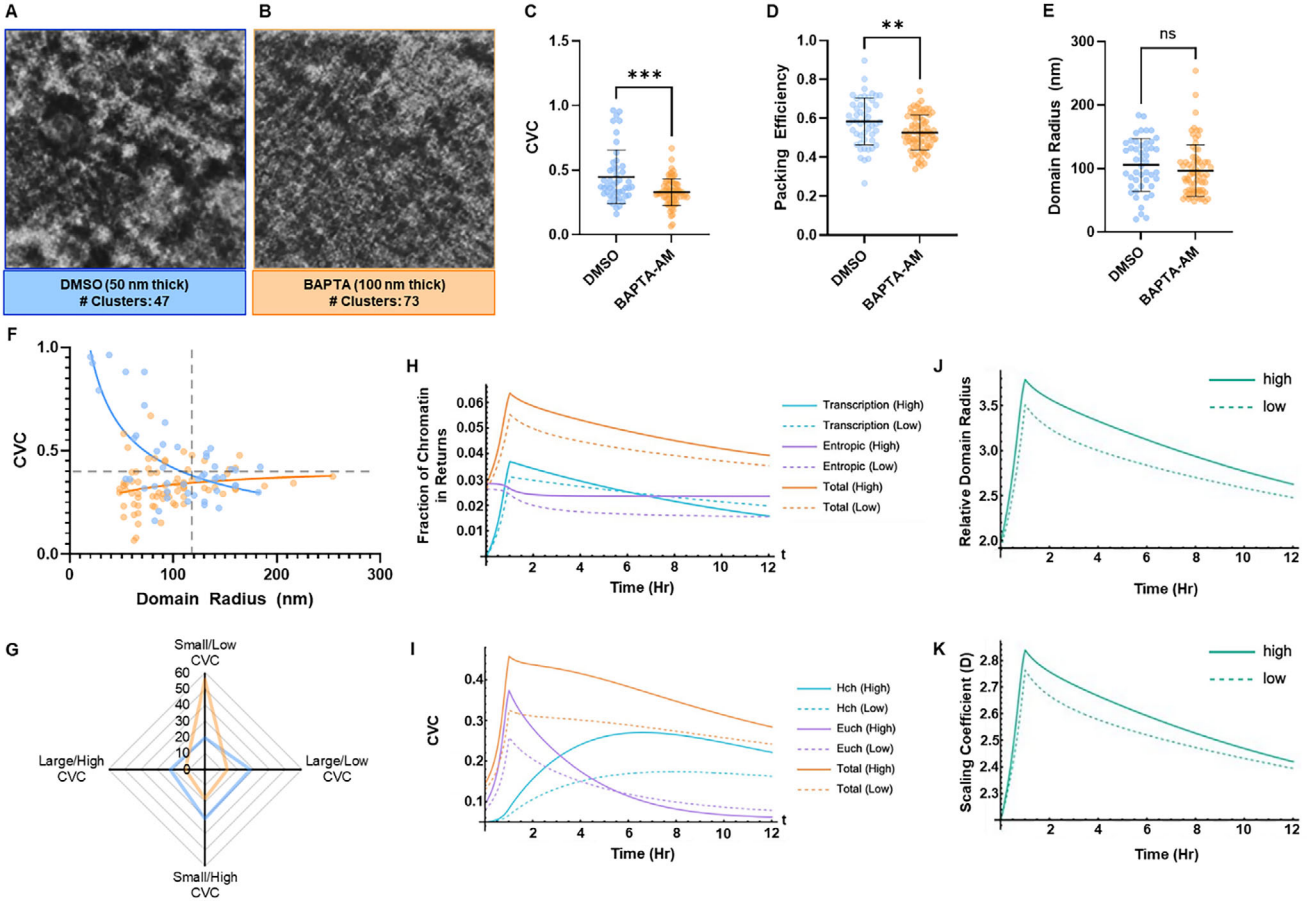
Ionic manipulation alters the number, size, and CVC of packing domains. (A) Representative tomogram from DMSO treated HCT116 cells compared to (B) HCT116 cells treated with BAPTA‐AM for 60 min to chelate intracellular divalent cations. (C‐E) In depth analysis of packing domains identified in ion chelated compared to DMSO treated control cells chromatin volume concentration (CVC) (C), packing efficiency (D), and size (E) in both conditions. (F) Examination of packing domains by size and CVC to characterize domain properties. (G) Quantification of transient domains (small size, low CVC), Mature domains (high CVC), and unstable/decaying domains (large size, low CVC) of cells treated with DMSO (blue) and chelators (orange) (n = 47 packing domains in DMSO; n = 73 packing domains in BAPTA‐AM). (H) Phenomenological model predictions of the effect of ion concentration on the number of observed loops (orange), entropic loops (purple), and transcription‐mediated loops (blue) over time after initiation of transcription. (I) Model predictions of the effect of ion concentration on CVC in total chromatin (orange), euchromatin (purple), and heterochromatin (blue) that comprise a packing domain over time after transcriptional initiation. (J‐K) Consequence of ions on domain radius (J) and scaling coefficient (D) (K) within domains demonstrating the increased domain maturation associated with increased ion concentration. Data are presented as the mean ± SD for each condition. Statistical significance was determined using a two‐tailed unpaired t‐test (^**^ *P* < 0.01, ^***^ *P* < 0.001).

ChromSTEM tomographic reconstructions of control (DMSO) and chelated (BAPTA‐AM) HCT116 cells revealed a striking reorganization of chromatin architecture at the nanoscale (Figure 2A,B). In control cells, chromatin is assembled into heterogeneous packing domains with a broad distribution of size and density. In contrast, magnesium chelation produced a visibly more fragmented and uniform chromatin landscape. Quantitative analysis confirmed these structural changes. Magnesium chelation significantly reduced CVC and packing efficiency (Figure 2C,D), indicating diminished domain compaction. This reduction in density was accompanied by an increase in the number of detected packing domains (46 in DMSO vs. 73 in BAPTA‐AM; Figure 2A,B), consistent with impaired maturation and stabilization of developing structures. Notably, domains <50 nm were not observed in chelator‐treated cells (Figure 2E,F), suggesting that divalent cations are required to stabilize the earliest nascent domain assemblies. Domains in the intermediate size range (∼50–100 nm) exhibited the most pronounced reduction in CVC, consistent with swelling of developing domains that have not yet acquired additional stabilizing reinforcements (Figure 2F). This pattern is consistent with reduced charge screening/neutralization weakening chromatin‐chromatin interactions necessary for efficient domain maturation [61, 62]. Consequently, loss of divalent cations resulted in the destabilization of maturing domains, which denature into smaller, lower CVC domains (Figure 2G). In contrast, domains larger than ∼120 nm showed relatively stable CVC between conditions, consistent with the idea that more mature domains possess additional structural support that reduces their dependence on divalent ions. The swelling of intermediate domains likely compensates for the disappearance of the smallest structures, resulting in minimal net change in overall radius distributions (Figure 2E) despite substantial alterations in packing density and domain stability [46, 47].

### 2.3 Phenomenological Modeling of Ion‐Mediated Chromatin Domain Dynamics

Motivated by the experimental observation that divalent cations regulate chromatin packing domain structure, we extended our previously established phenomenological model [16] to incorporate the effects of nuclear ions on PD formation and maturation. In the updated model, we describe the temporal evolution of chromatin packing architecture under dynamic transcriptional and biophysical conditions with ionic conditions captured through corresponding changes in the parameters governing polymer folding, enzymatic accessibility, and transcriptional feedback. Chromatin is represented as a flexible polymer confined to a sub‐volume of the nucleus, where it self‐organizes into PDs with a distribution of packing states related to function. In the model, each PD follows a power‐law polymer scaling law, in which the local chromatin volume fraction decays radially from the PD center. This framework allows investigation of the contribution of both molecular crowding and spatial organization across hierarchical length scales involved in domain formation, maintenance, and decay.

To investigate the role of ions, we systematically modified model parameters to reflect known biophysical consequences of elevated divalent cation concentrations (Materials and Methods). Under high divalent‐ionic conditions, there is enhanced charge screening, enhanced activity of topoisomerases and RNA polymerase, and a higher likelihood of entropic stabilization by salt bridge formation. We model these effects by increasing the initial packing efficiency to represent enhanced charge screening, increasing the rate of transcriptional returns, elevating the topoisomerase scaling factor to capture magnesium's catalytic role in DNA relaxation, and reducing the crosslinking memory threshold to simulate stronger entropic stabilization. In this framework, divalent ion contributions are explored as a physiochemical variable that modulates chromatin packing through charge neutralization and phosphate bridging, thereby stabilizing local density pockets and facilitating domain maturation.

To evaluate the effects of ionic conditions on chromatin architecture, we calculated chromatin domain kinetics under high and low ion concentrations during active transcription and following its cessation. Domains were first allowed to form and evolve for one hour in the presence or absence of elevated ions, after which transcription was simulated to turn off to isolate the contribution of ions to domain maintenance (Figure 2H). Under high‐ion conditions, the total chromatin engaged in loop returns increased rapidly during transcription, primarily driven by transcription‐mediated returns. After transcription was terminated, elevated ion concentrations preserved packing domains more effectively, primarily through the stabilization of entropic returns, passive loop contacts arising from polymer compaction, and charge‐mediated stabilization, even as transcription‐mediated returns diminished. Conversely, low‐ion conditions resulted in slower domain growth and a pronounced loss of entropic returns after transcriptional inhibition, indicating that ions play a critical role in domain formation, maturation, and the preservation of structural memory within chromatin.

Consistent with these effects, model predictions of the CVC of euchromatic regions increased more rapidly under high‐ion conditions, producing a higher total CVC and more stable packing geometry (Figure 2I). Upon transcriptional arrest, CVC remained elevated for several hours in high‐ion environments, indicating that although the loss of transcriptional boundary forces would normally and eventually lead to domain degradation, elevated ions preserve chromatin packing and support gradual maturation into more heterochromatic configurations. These effects were less pronounced in low‐ion conditions, in agreement with ChromSTEM measurements (Figure 2C). Moreover, the model predicts that ion abundance significantly enhances domain radius and packing scaling (D) (Figure 2J,K), consistent with the emergence of mature, compact chromatin structures.

Together, these calculations demonstrate that ionic charge screening not only accelerates the formation of nascent domains during active transcription but also sustains their structural integrity once polymerase is no longer acting as an active boundary element. This phenomenological model provides a mechanistic framework that unites ionic regulation with the physical organization of chromatin, linking the observed experimental transformations in ChromSTEM and live‐cell imaging to the fundamental principles of domain formation, maturation, and transcriptional stability.

### 2.4 Magnesium Regulates the Interplay of Active RNA Polymerase With Heterochromatin Cores

Although divalent cations are critical for maintaining chromatin structure, their role in shaping the biophysical properties of methylated nucleosomes is poorly characterized. Prior work has shown that prolonged extracellular chelation (∼24 h) alters the activity of histone methyltransferases [57]. However, the targeted depletion and doping of intracellular and intranuclear ionic conditions on histone remodeling and its effect on chromatin domains has not been studied. Given our findings of the effect of magnesium manipulation on chromatin packing domains in ChromSTEM, we hypothesized that magnesium is crucial for organizing chromatin into packing domains. Specifically, in prior work, it was demonstrated that heterochromatin acts as a space‐filling scaffold that supports RNA transcription near the periphery [16, 21, 63]. This is likely due to the competition that results between the diffusibility of the reactant species (transcription factors, polymerases) and the stabilization of intermediate complexes [7, 11, 16, 22]. Consequently, the modulation of domains on ChromSTEM (Figure 2) is predicted to destabilize the coherent positioning of RNA polymerase II in relation to domain cores made of heterochromatin (H3K9me3). To investigate this, we utilized stochastic optical reconstruction microscopy (STORM) imaging on control, divalent cation chelated, and magnesium‐doped samples. STORM imaging allows us to investigate changes in high‐density heterochromatin marks (H3K9me3) associated with domain cores with active transcription (RNA Pol II).

**FIGURE 3:**
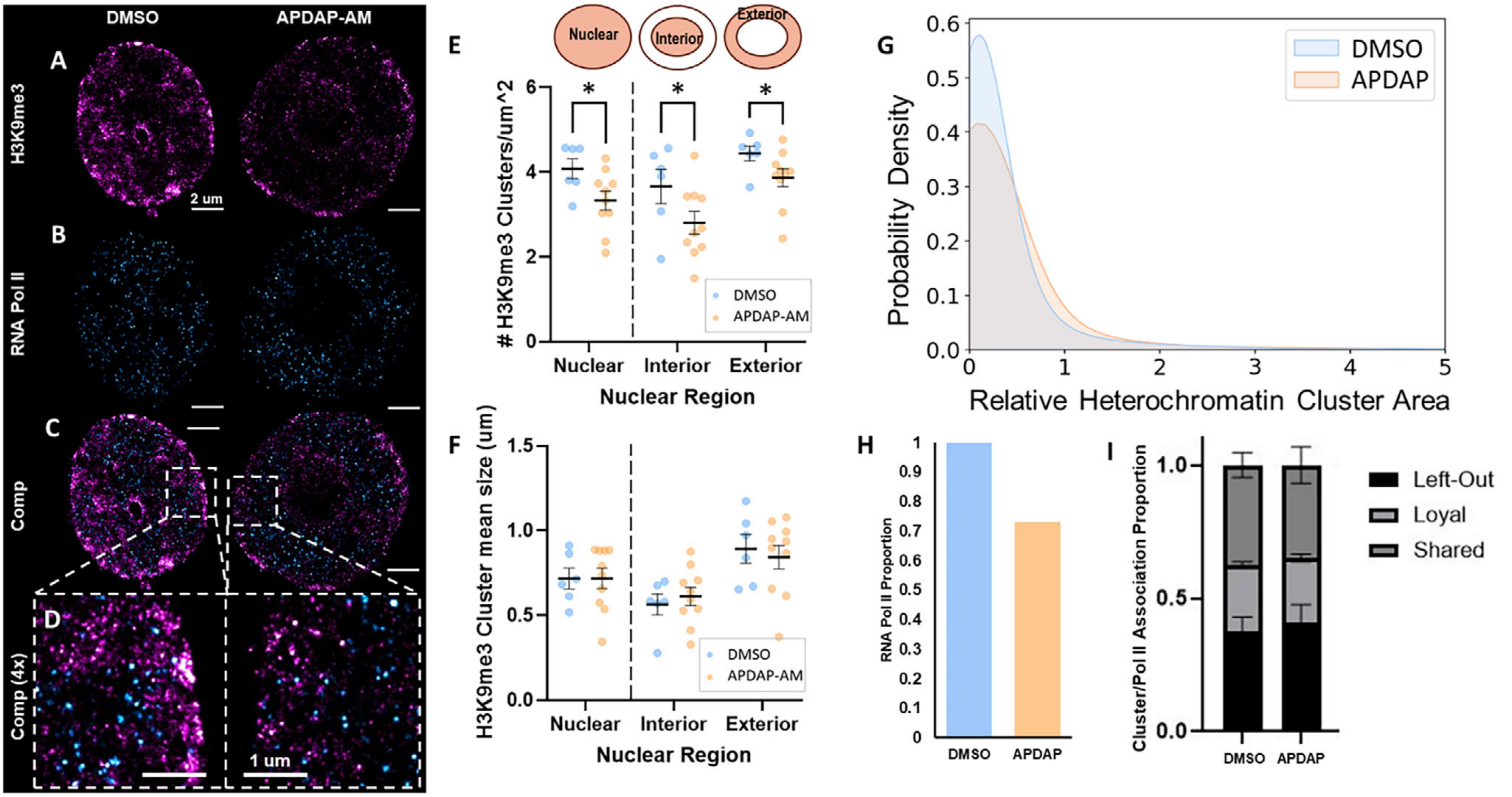
Divalent cations regulate the number, size, and localization of H3K9me3 heterochromatin clusters and RNA Pol II association. (A‐D) Representative multiplexed STORM images demonstrating the spatial localization of H3K9me3 (magenta, A) and active RNA polymerase II [Serine‐2 phosphorylated (Pol2‐PS2), blue, B] labeled in the nuclei of HCT116 cells treated with APDAP‐AM for 60 min (C). (D) 4x zoomed in regions of indicated compiled images to visualize the change in H3K9me3 density at the periphery, decrease in Pol2‐PS2 quantity, and increased instances of Pol2‐PS2 staining not associated with H3K9me3 clusters. (E‐F) Quantification of the effect of divalent chelation on H3K9me3 quantity corrected by the nuclear area (E) and H3K9me3 cluster size (F) for the whole nucleus (nuclear), center of the nucleus (interior), and outer 1 µm of the nucleus (exterior) (Data are the average of all identified H3K9me3 clusters in each processed image, N = 9). (G) Kernel density estimation of relative H3K9me3 heterochromatin cluster area (normalized to r = 80 nm) upon divalent chelation demonstrating a global loss of domain interiors. (H) Quantification of Pol2‐PS2 demonstrating a global loss in transcription upon chelation. (I) Quantification of the association between Pol2‐PS2 and H3K9me3 cores normalized to the total amount of Pol2‐PS2 in each condition demonstrating that less RNA Pol II is near H3K9me3 cores (loyal and shared) and more is not associated with any heterochromatin domains (left‐out). Data are presented as the mean ± SD for each condition. Statistical significance was determined using a two‐tailed unpaired t‐test (^*^ *P* < 0.05).

Although H3K9me3 is associated with constitutive heterochromatin that is transferred across cell division, we observed the rapid loss of high‐density H3K9me3 aggregation after treatment with chelators, especially at the nuclear periphery where heterochromatin‐rich regions dissipate within 1 h (Figure 3A–D, Figure S3). This finding was consistent with the transformation to domain cores observed on ChromSTEM imaging. Contrasted with Hi‐C evidence showing that A/B compartments are resilient to swelling and chelation events [64], this finding suggest that divalent cations are crucial for the maintenance of chromatin domain geometry rather than being maintained by the strength of the segment connectivity. Within the nuclear interior, we similarly observed a decrease in the number of H3K9me3 clusters across the nucleus with divalent cation chelators (Figure 3A,D,E). Interestingly, the average size of H3K9me3 clusters does not change upon treatment with APDAP‐AM (Figure 3F), this is likely due to numerous remaining PDs swelling under chelation (Figure 3G), but some of those domains swell to a point where they dissociate, forming smaller H3K9me3 structures. This aligns with ChromSTEM findings showing that maturing domains, which should correlate to heterochromatin‐rich regions, decrease in response to divalent cation chelation with some large low CVC domains remaining. Functionally, this structural remodeling is accompanied by a marked reduction in active transcription upon divalent cation chelation (Figure 3H). Importantly, among the remaining RNA polymerase II foci, their spatial distribution relative to H3K9me3 domains is largely preserved, with only a modest increase in polymerase signals located outside heterochromatic clusters (Figure 3I). These findings suggest that the decrease in polymerase upon chelation primarily reflects the loss of mature H3K9me3‐enriched domains, while the stabilized domains that persist continue to support similar transcriptional activity. Together, these results indicate that divalent cations selectively stabilize maturing chromatin domains, and that their depletion preferentially disrupts domain geometry in a spatially and maturation‐dependent manner, thereby reshaping transcriptional capacity across the nucleus.

### 2.5 Nuclear Cation Concentration Regulates Chromatin Domains In Live Cells

**FIGURE 4:**
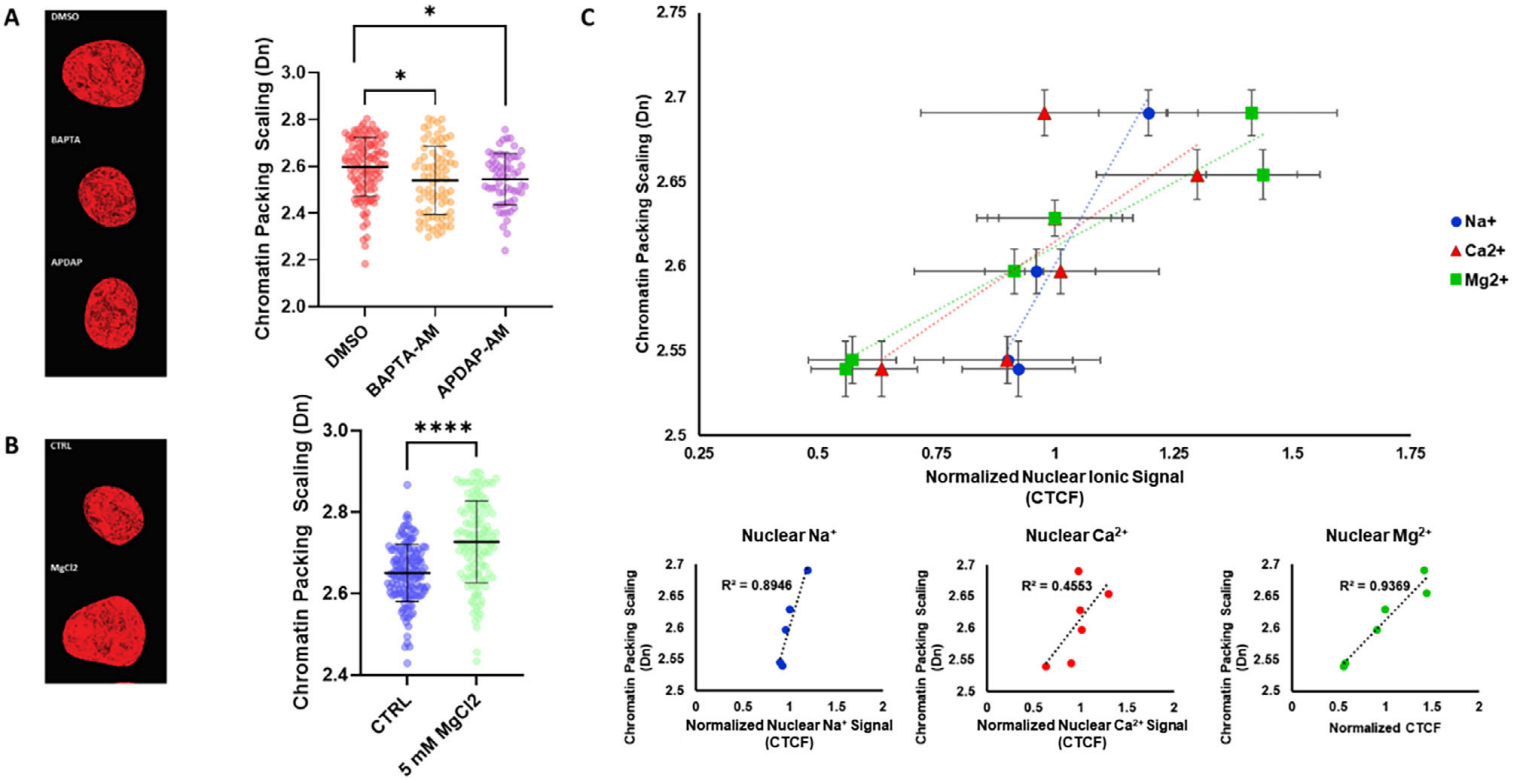
Nuclear Mg^2+^ concentration drives chromatin remodeling in live cells. (A) Live‐cell PWS nanoscopy of BAPTA‐AM and APDAP‐AM chelated HCT116 cells at 1 h demonstrating a decrease in chromatin packing scaling, D_n_, consistent with decaying packing domains (n = 115; mean ± SD). (B) Live‐cell PWS nanoscopy of MgCl_2_ treated HCT116 cells at 1 h demonstrating an increase in chromatin packing scaling, D_n_, consistent with domain formation and maturation (n = 150; mean ± SD). (C) Quantification of the average CTCF for Na^+^, Ca^2+^, and Mg^2+^ ions in the nucleus of HCT116 cells treated with divalent cation chelators and MgCl_2_ plotted versus the average D_n_ for each respective treatment condition. The linear fit of Na^+^, Ca^2+^, and Mg^2+^ indicates a strong monotonic correlation between nuclear Mg^2+^ ions and D_n_. Data are presented as the mean ± SD for each condition. Statistical significance was determined using one‐way ANOVA with Tukey's post‐hoc test (^*^ *P* < 0.05, ^****^ *P* < 0.0001).

Having demonstrated that manipulation of ions predictably regulates chromatin packing domain structure and function in situ, we next investigated the consequence of intranuclear ionic concentrations on the structure of chromatin packing domains in live cells. Furthermore, temporal variations in scattering were used to measure chromatin mobility. Fractional moving mass (FMM), calculated from the product of the mass of the moving macromolecular cluster and the volume fraction of moving mass, quantifies the physical and dynamic properties of chromatin, as not all motion within the cell is diffusive [65]. Although each of these parameters describes how the physical assembly of the genome intersects with molecular function, we initially focus on the change in D as it (1) describes the polymeric folding behavior of chromatin within domains, (2) regulates transcriptional patterns, (3) is increased in malignancy and chemoevasion, and (4) plays a pivotal role in phenotypic plasticity [7, 66, 67, 68]. Utilizing dual‐partial wave spectroscopic (PWS) nanoscopy, we measured in real‐time the change in D_n_ (the amount of chromatin organized into packing domains within the nucleus), and fractional moving mass (FMM, the magnitude of chromatin evolving in time) in response to ion chelation. We hypothesized that divalent ions play a central role in regulating chromatin structure through ion condensation and DNA‐phosphate‐Mg^2+^ bridging, thereby mitigating excess electrostatic repulsions within packing domains and preserving domain integrity. Consequently, we anticipate that removal or chelation of divalent ions would compromise domains, resulting in a rapid decrease of D_n_ values in live cells. Here, an increased D_n_ corresponds to an increased likelihood of chromatin organizing into packing domains and vice versa (Figure 4A,B) [8, 68]. It is crucial to note that D_n_ values are bounded between 2 and 3, reflecting the filling in power‐law space of the chromatin chain (the packing statistics of the entire genome, 6.02*10^9^ bp). Therefore, a 2–5% change in D_n_ represents a significant transformation in chromatin packing across a cell population as it reflects reposition of contents on the order of the entire protein‐coding contents of the genome. Consistent with a reduction of chromatin packing domains, chelation of divalent cations with BAPTA‐AM and APDAP‐AM for 60 min showed a decrease in D_n_ to 2.23% ± 0.63% and 2.02% ± 0.53%, respectively, from a baseline of 2.6*** (standard error of the means (SEM)) (Figure 4A). Next, we investigated if the reverse process, increasing nuclear cations, would result in more domain formation. To test this, we delivered divalent cations (Mg^2+^) via the addition of a MgCl_2_ solution as above and utilized PWS nanoscopy to measure the change in domain formation. Treatment with 5 mM MgCl_2_ increased domain formation occurred with extracellular delivery of Mg^2+^ for 60 min which resulted in a significant increase in D_n_ to 3.08% ± 0.48% (Figure 2B).

Given the observed heterogeneity in chromatin states within a cellular population, we next investigated whether these variations correlated with intranuclear ionic concentrations. To test this hypothesis, we measured both D_n_ and relative ionic concentrations (Na^+^, Ca^2+^, and Mg^2+^) simultaneously in control cells, ion‐chelated cells, and Mg^2+^‐loaded cells. Remarkably, intranuclear ionic conditions strongly correlated with D_n_ across populations even at short treatment timescales (60 min, Figure 4C). Of these conditions, Mg^2+^ concentrations displayed the most robust linear relationship with D_n_, indicated by an R^2^ of 0.94, while Ca^2+^ and Na^+^ showed weaker correlations across the population (Figure 4C). In the context of the overall hypothesis that ionic conditions are necessary to maintain domain stability, these findings are consistent with the role of cations, particularly magnesium, in associating with the DNA phosphates to stabilize charge and promote chromatin packing [38]. Although Mg^2+^ and Ca^2+^ share the same valence, their physicochemical properties differ substantially. The effective ionic radius of Ca^2+^ varies depending on coordination number (100‐126 pm), compared to ∼72 pm for Mg^2+^, and Ca^2+^ possesses a weaker hydration shell. In contrast, Mg^2+^ is smaller, more strongly hydrated, and exhibits higher charge density, properties that favor stable coordination with phosphate groups and promote charge shielding and bridging interactions. Consistent with their distinct physiological roles, Ca^2+^ is maintained at low, transient concentrations and functions primarily as a signaling ion. These distinctions may account for why Ca^2+^ ions were not as strongly correlated with D_n_. While Ca^2+^ ions are required for the initiation of chromatin relaxation in T‐cells [69], they have also been shown to increase chromosome compaction in HeLa cells undergoing mitosis under elevated salt conditions [45]. This suggests that while various species of divalent cations can impact chromatin organization, Mg^2+^ ions exert a stronger effect on chromatin packing domains.

### 2.6 Nuclear Cation Concentrations Rapidly and Nonlinearly Modulate Domain Structure

**FIGURE 5:**
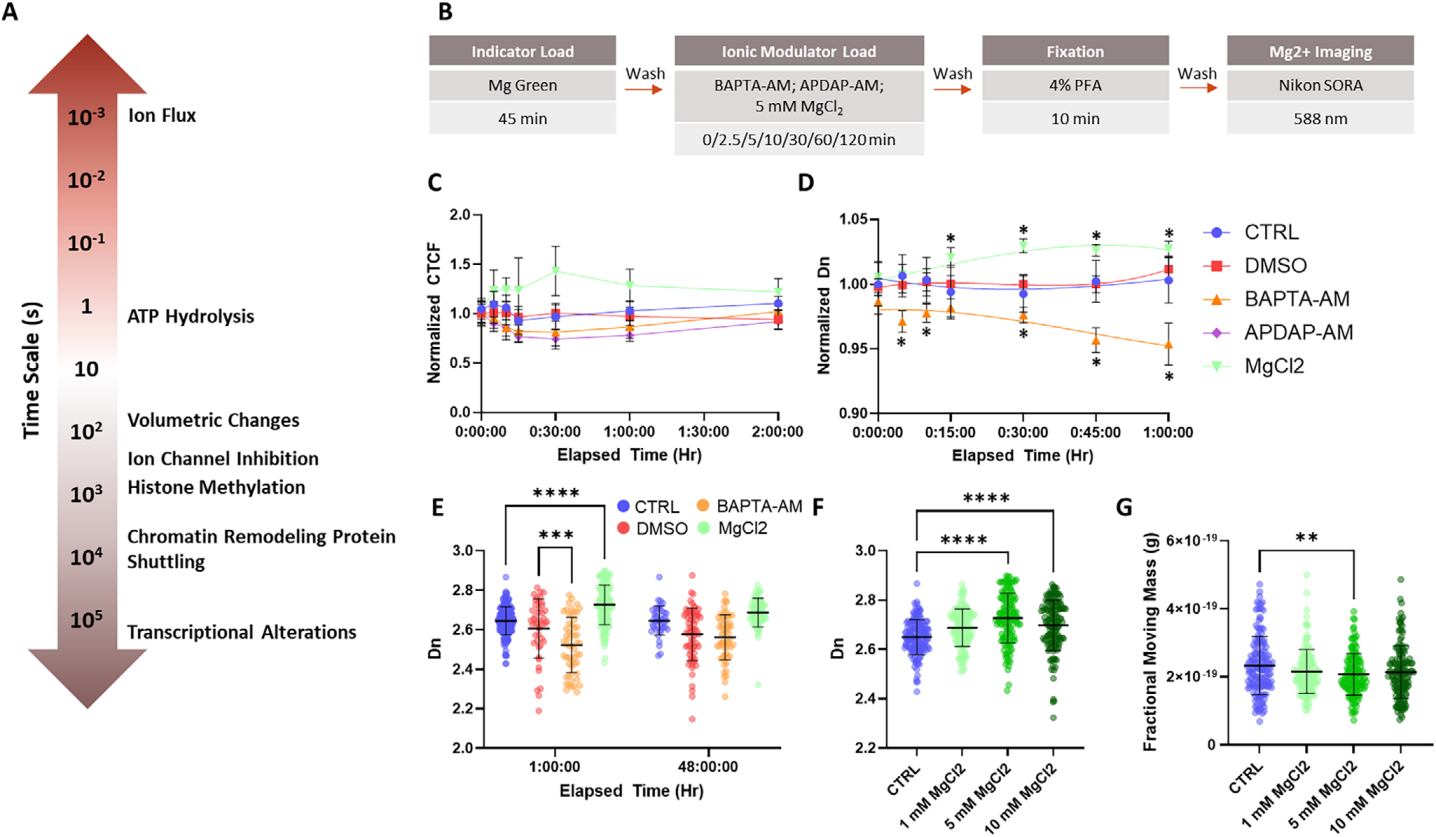
Nuclear Mg^2+^ induces rapid, sustained, and dose‐dependent modifications in chromatin packing. (A) Depiction of the time scales of various ion‐linked alterations in cellular processes. (B) Outline of protocol for staining and imaging HCT116 cells stained with Mg^2+^ ion indicator. (C) Nuclear Mg^2+^ ion concentration determined from confocal imaging of Magnesium Green stained HCT116 cells fixed at various timepoints up to two hours post‐treatment with divalent cation chelators and MgCl_2_ (n = 10; mean ± SEM). (D) Live‐cell nanoscopy tracking the time‐evolution of packing domains in individual cells treated with divalent cation chelators and MgCl_2_ for one hour (n = 30; ± SEM). (E) PWS imaging of HCT116 cell populations treated with ion chelators or MgCl_2_ for 1 h and 48 h, indicating that ionic regulation is a transient process (n = 150 (CTRL), n = 100 (All other conditions); mean ± SD. (F‐G) Live‐cell nanoscopy of HCT116 cells treated with increasing concentrations of MgCl_2_ at one hour demonstrating an increase in D_n_ (F) and a decrease in FMM (G) consistent with domain compaction and nascent domain synthesis (n = 150; mean ± SD). Statistical significance was determined using one‐way ANOVA with Tukey's post‐hoc test (^*^ *P* < 0.05, ^**^ *P* < 0.01, ^***^ *P* < 0.001, ^****^ *P* < 0.0001).

Although 1 h is short compared to the time scale of cellular replication, ionic interactions are crucial for multiple rapidly evolving processes such as ATP hydrolysis, protein shuttling, DNA replication, enzymatic modification of histones, and RNA polymerase function. To date, studies on the temporal evolution of the genome have identified that loop formation is a relatively stable, albeit infrequent, event that occurs ∼2 times within a 12‐h cycle, with DNA remaining in this loop configuration for 20–45 min [70]. Likewise, at long‐time scales (>30 s) and across the whole genome, chromatin has been reported to exhibit solid‐like behavior that is resistant to rapid transformation [10]. We have previously demonstrated that chromatin domains rapidly respond to redox conditions within the cell, with nuclear‐wide collapse of domains occurring at the timescale of minutes with disruption of the mitochondrial membrane potential [67]. Therefore, to understand if ionic depletion could behave similarly to redox disruption, we measured the transformation of ion levels and chromatin structure on timescales ranging from seconds to minutes. By investigating the temporal response of chromatin to ionic modulation, we can decouple the impact of direct ionic interactions (seconds‐minutes) from other responses that would take longer to affect chromatin conformation (hours‐days) (Figure 5A,B). Previous studies demonstrated that BAPTA‐AM significantly reduces the concentration of Ca^2+^ within cells in 2–5 min [71, 72]. We validated this result and further explored the timescale at which APDAP‐AM and ion loading significantly alter intracellular ion concentrations (Figure 3C). Treatment with 10 uM of BAPTA‐AM or APDAP‐AM decreased the nuclear Mg^2+^ ion concentration by 15% within 5 min of incubation but, unexpectedly, nuclear Mg^2+^ levels returned to baseline within 2 h despite continued chelator incubation (Figure 5C). In contrast, incubating cells with 5 mM MgCl_2_ significantly increased nuclear magnesium ion concentration by 24% within 5 min of treatment, and elevated nuclear Mg^2+^ levels returned to control levels within 2 h (Figure 5C). This is consistent with the previously reported effect of chelators, such as BAPTA‐AM, which failed to continuously abolish intracellular Ca^2+^ ions in response to stimulation within 3–5 h [72], and that prolonged MgCl_2_ treatment does not produce a stable change in cellular Mg^2^^+^ [57]. Together, these results reinforce that ion flux in cells is inherently a well‐regulated process.

Consistent with ultra‐rapid modulation of chromatin domains comparable to the perturbation of intracellular redox state [73], ion chelators and divalent cation donors produced a rapid transformation in D_n_. Specifically, within 5 min (the timescale for the intracellular transformation of ion levels Figure 5C), magnesium chelation resulted in the suppression of packing domains (Figure 5D). Furthermore, Mg^2+^ delivery increased D_n_ after 15 min (Figure 5D). Consequently, these findings are in line with the direct effect of magnesium ions on the stabilization of chromatin domains in living, interphase nuclei at timescales greatly exceeding those produced by disruption of loop extrusion or nucleosome modification [70, 74]. At the time scale of cellular replication (24‐48 h), continuous removal of intracellular magnesium by chelation did not maintain the downregulation of packing domains. Similarly, we observed that domains re‐formed to baseline levels after continuous application of magnesium ions (Figure 5E). This can be explained because the reaction kinetics of chelators decrease over time [72], and cells can mobilize adaptive responses, such as ion channels (TRP channels, Mg^2+^ transporters), to restore baseline ion levels [75, 76, 77].

Next, we investigated whether magnesium delivery induces a dose‐dependent transformation of domain structure within short timescales (60 min). To test this, we treated cells with increasing concentrations of MgCl_2_ (1, 5, 10 mM) and found that domain formation was indeed a dose‐responsive effect, with the highest D_n_ observed with 5 mM Mg^2+^ (Figure 5F). To investigate the temporal effect of ionic modulation on chromatin organization, HCT116 cells were treated with ionic modulators, and individual cell nuclei were tracked in real‐time using live‐cell Dual Partial Wave Spectroscopic (Dual‐PWS) nanoscopy [56]. Interestingly, increasing the nuclear Mg^2+^ ions decreases the average FMM within the nucleus (Figure 3G). Initially, this result seemed to contradict the predicted consequence of increasing the nuclear divalent cation content, further compacting chromatin, and therefore increasing the mobile mass of chromatin domains (as they are comprised of more chromatin). However, if new domains are being formed in the presence of Mg^2+^, this will produce many small domains with small mobile mass and will therefore decrease the average FMM.

### 2.7 Cation Modulation Alters Gene Transcription and Decreases Transcriptional Plasticity

Chromatin domain geometry plays a critical role in transcriptional regulation [7, 22, 66, 73, 78]. As demonstrated by ChromSTEM findings, the removal of divalent cations, particularly Mg^2+^, alters chromatin PDs, redistributing the hetero‐ and euchromatic regions and reshaping the transcriptional landscape. Importantly, this destabilization is not uniform across all PDs. ChromSTEM analysis indicates that nascent and weakly stabilized domains not held together by methylation and cross‐linking enzymes are preferentially disrupted (Figure 2). Consistent with this observation, phenomenological modeling predicts that reduced ionic strength lowers the efficiency of nascent domain formation while accelerating structural decay (Figure 2). Rather than simply increasing chromatin accessibility, this reorganization compromises the structural framework required to maintain optimal transcriptional microenvironments, thereby altering RNA polymerase positioning and activity across the genome. This phenomenon is particularly relevant in cancer cells, which exhibit exceptional transcriptional plasticity and dynamic chromatin remodeling that enable adaptation to stress and therapeutic resistance [7, 9]. In highly plastic systems such as cancer cells, the inability to form and stabilize new domains limits adaptive reprogramming. Consistent with this framework, RNA‐seq principal component analysis indicates that chelation of Mg^2+^ has a substantial impact on the HCT116 transcriptome (Figure S4A). Analysis of volcano plots visualizing gene expression changes and the 1,000 most differentially expressed genes demonstrated that chelation of divalent cations significantly increases and decreases the expression of multiple genes (Figure 4A,B; Figure S4). This suggests that removing divalent cations disrupts chromatin domain stability, thereby bi‐directionally altering transcription.

**FIGURE 6:**
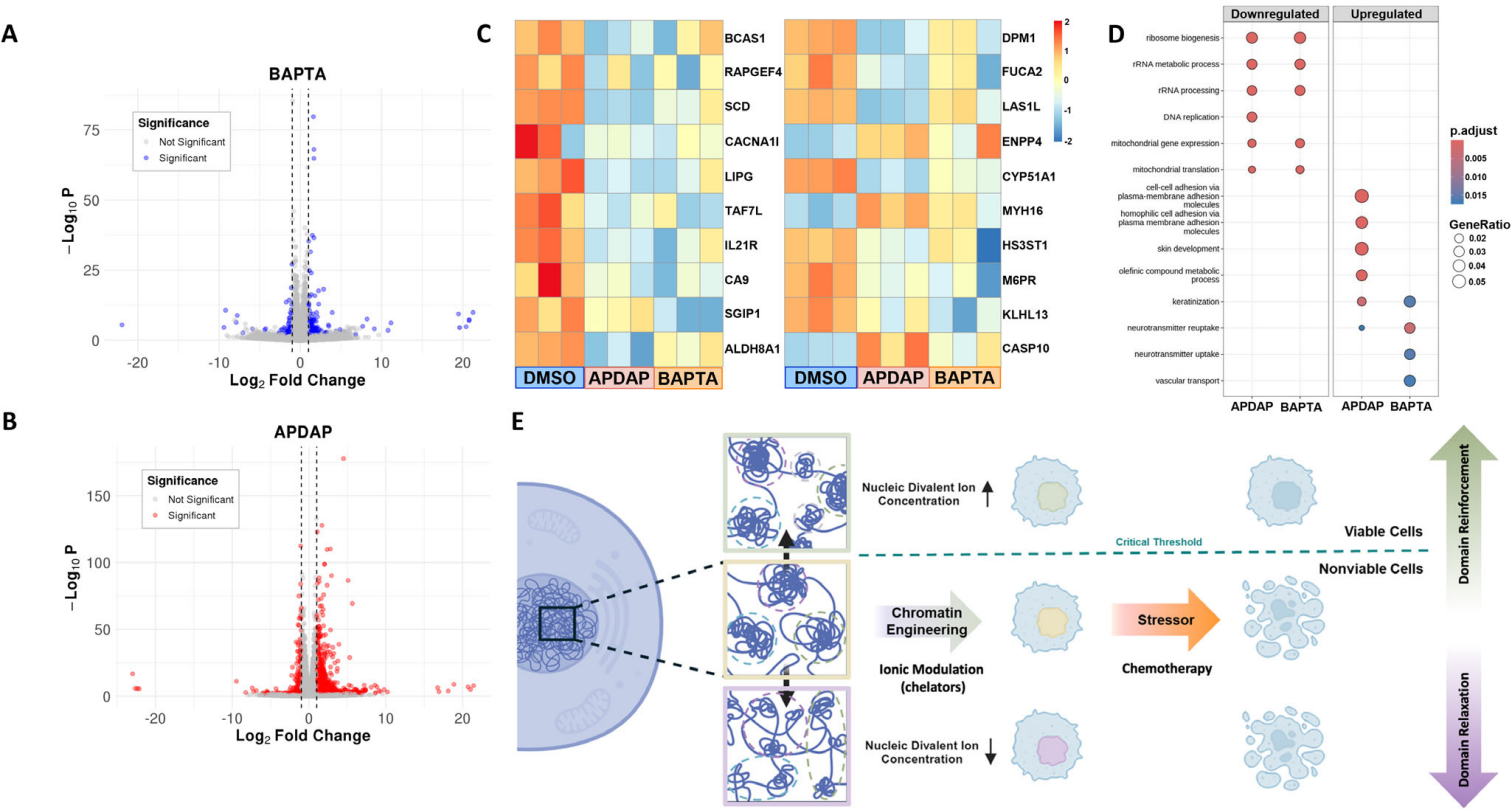
Divalent cation depletion alters gene transcription and transcriptional divergence in HCT116 cells. (A‐B) Volcano plots of the significantly up‐ and down‐regulated genes identified using RNA‐seq performed on HCT116 cells treated with divalent cation chelator BAPTA (A) and APDAP (B) indicating bi‐directional regulation of transcription. (C) RNA‐seq heat map of differentially expressed genes upon treatment with divalent cation chelators compared with DMSO treated controls. (D) Top down‐ and up‐regulated GO processes in HCT116 cells treated with APDAP and BAPTA. (E) Depiction of the role of chromatin engineering in driving transcriptional divergence in HCT116 cells.

This disruption of chromatin PDs not only influences transcriptional patterns but also affects cellular plasticity, particularly in cancer cells. In the context of PD evolution, perturbation of the ionic balance shifts the equilibrium of chromatin density in transcriptionally active regions, thereby disrupting optimal transcriptional conditions. Excessive formation of chromatin packing domains has been implicated as a potential mechanism of tumorigenesis, increased cellular plasticity, and chemoevasion in cancer cells [7, 9, 79, 80]. For a cell with a higher average D_n_, the probability of any given gene within the cell being in a packing domain increases, and therefore, the likelihood that a gene is in optimal physiochemical conditions for transcription increases as well. We predicted that Mg^2+^ ion chelation would decrease cancer cell plasticity by preventing gene expression in stemness and cell survival pathways since BAPTA‐AM and APDAP‐AM destabilize chromatin packing domains. Bulk RNA sequencing performed on DMSO, 1‐h BAPTA‐treated, and 1‐h APDAP‐treated cells confirmed that Mg^2+^ ion chelation rapidly downregulates several key genes that facilitate cancer cell survival. This includes ALDH3A1, a gene that encodes for an aldehyde dehydrogenase enzyme utilized by cancer stem cells for resilience to oxidative stress (Figure 6C). Interestingly, both chelators downregulated CACNA1I, a gene that encodes a subunit of voltage‐gated calcium channels, and CA9, which encodes a tumor‐associated carbonic anhydrase isoform (Figure 6C). Both treatments also upregulated genes such as ENPP4, MYH1B, and CASP10. Notably, ENPP4 encodes for an enzyme involved in ADP and ATP production, while CASP10 produces one of the caspases activated by death receptors in the extrinsic apoptosis pathway. While some genes had a similar change in expression with both chelators, genes such as ALDH3A1 and CASP10 showed remarkable differences between APDAP and BAPTA treatment. These gene‐specific variations suggested mechanistic differences in cellular response based on the chelated ions, as BAPTA‐AM binds both nuclear Ca^2+^ and Mg^2+^ ions, whereas APDAP‐AM preferentially binds to nuclear Mg^2+^ ions (Figure 1C,D).

We next sought to determine whether gene‐specific changes reflect pathway alterations in chelator‐ treated cells using gene ontology (GO) enrichment analysis. Our analysis confirmed that the major effect of Mg^2+^ ion chelation is downregulation, which is apparent in the gene count for each significantly enriched term (between 50–150 genes for downregulated terms versus 2–5 for upregulated terms) and the adjusted enrichment p‐values (downregulation adjusted p‐value: ∼1 × 10‐11 – ∼5 × 10‐12; upregulation adjusted p‐value: ∼0.04 – ∼0.01; Figure 6D). The specific downregulated terms included biological processes associated with RNA processing, ribosome biogenesis, ATP production (cellular respiration, aerobic respiration, ATP biosynthesis), and nucleotide biosynthesis (Figure 6D). Similarly, both treatments downregulated molecular functions associated with DNA/RNA catalysis, ATP hydrolysis, RNA pol II binding, and cadherin binding (Figure S4D). Upregulated terms were biosynthetic processes, cholesterol homeostasis, and lipid metabolism, although the enrichment effect was minimal (Figure 6D).

Lastly, we calculated the sensitivity of gene expression to changes in chromatin packing domains to evaluate whether the transcriptional differences between the chelators can be explained by their impact on domains. Within the framework of the chromatin packing molecular crowding (CPMC) model, changes in D_n_ directly modulate the local crowding environment that governs enzyme accessibility and transcriptional efficiency. As D_n_ increases, there is a greater portion of chromatin that is incorporated into structured domains, thereby increasing the amount of chromatin that exists in the optimal physicochemical “goldilocks” zone for transcription. Therefore, we investigated how chelation‐mediated destabilization of chromatin packing domains impacts transcription by calculating gene expression sensitivity (*Se*) as *Se* = Δln(E)/Δln(D_n_), where E is the average transcript‐per‐million (TPM) expression value of a group of genes for untreated control cells (E_1_) and for 1‐h treated cells (E_2_). The resulting value for *Se* therefore quantifies how much of the change in gene expression is due to the change in chromatin packing domains caused by chelation. We separated genes by their expression value in the control group, as we have previously seen that the initial expression level of a gene influences the amount of change it experiences because of a domain‐altering treatment. Both chelators showed a similar trend in that genes that are under expressed in the control group (negative x‐axis) were suppressed further with chelation (negative *Se*), and overexpressed genes were upregulated (Figure S4C). One interesting phenomenon is that initially strongly under expressed genes are not suppressed as strongly as moderately under expressed genes (ln(E_Control_/E_Control_) of ∼‐4 versus ∼‐1) upon divalent cation chelation (Figure S4C,D). Additionally, while BAPTA‐AM had a modest effect (‐5 < Se < 5), APDAP‐AM caused much more extreme changes in expression (‐15 < Se < 10; Figure S4C) which reflected the gene‐specific results (Figure 6C). These findings show that while cation chelation causes many global transcriptional changes, the variation between Ca^2+^ and Mg^2+^ can create different magnitudes of impact on individual gene patterns at short‐time scales. Based on our results, we hypothesized that destabilizing packing domains with cation chelation could prevent chemoevasion in cancer cells (Figure 6E).

### 2.8 Modulation of Chromatin Domains Can Predictably Reprogram Cancer Cells

**FIGURE 7:**
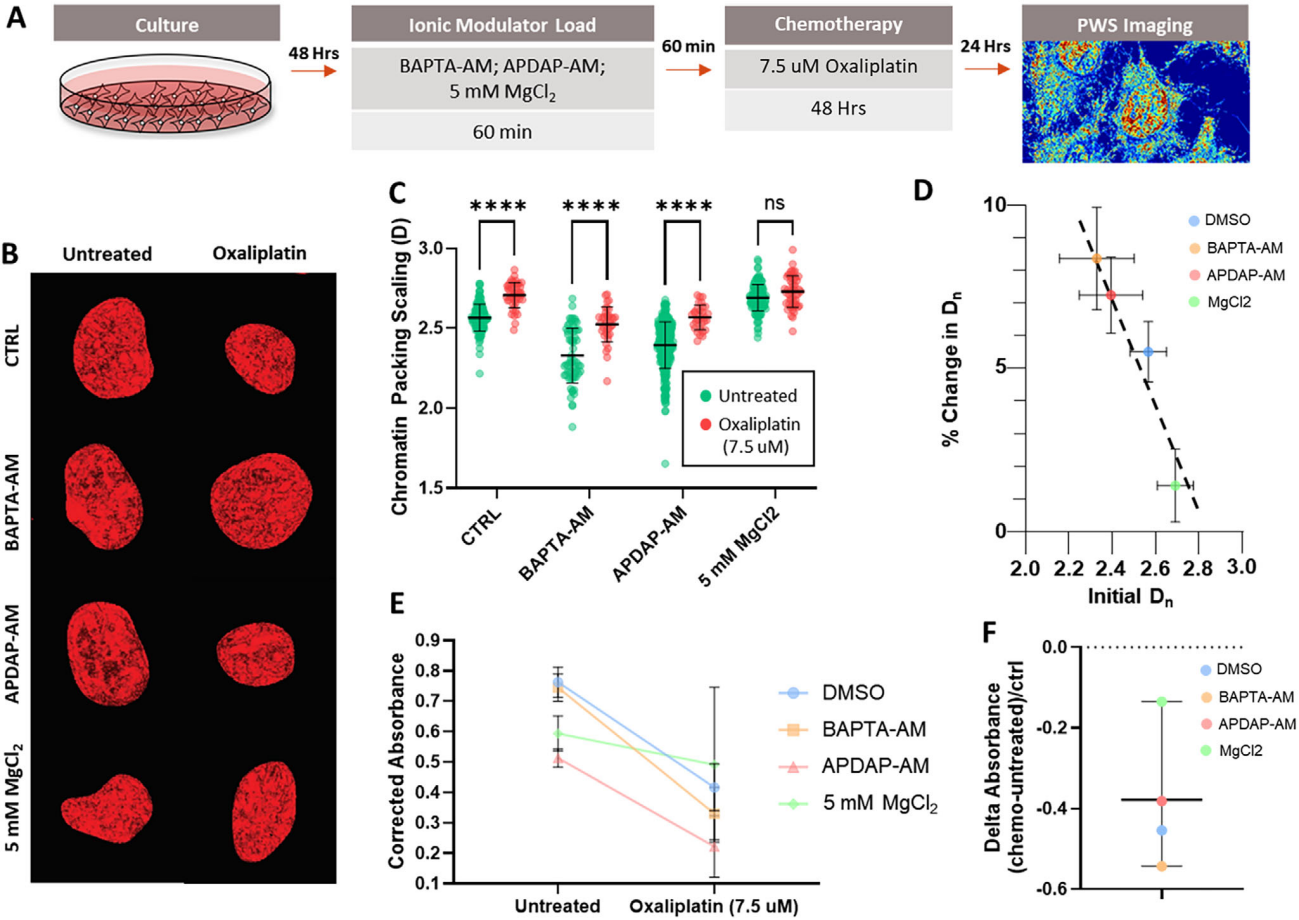
Application of ionic chromatin remodeling for synergistic lethality in HCT116 cells. (A) Schematic of the experimental workflow to assess synergistic lethality in HCT116 cells treated with divalent cation chelators or extracellular MgCl_2_ followed by 7.5 µM oxaliplatin, targeting chromatin packing domains. (B, C) Live‐cell nanoscopy of HCT116 cells treated with ion chelators prior to oxaliplatin exposure shows increased chromatin packing scaling (D_n_) in response to chemotherapy, while treatment with MgCl_2_ increased initial D_n_ with limited change in response to chemotherapy (n = 150–300 cells; mean ± SD). (D) Scatter plot of average initial D_n_ after chelator or MgCl_2_ treatment versus changes in D_n_ after 48 h of oxaliplatin exposure, revealing an inverse correlation between initial chromatin state and chemotherapy‐induced packing changes. (E, F) Cell viability was measured by PrestoBlue assay (absorbance background‐corrected). Cells pre‐treated with chelators exhibit reduced viability following oxaliplatin exposure, with BAPTA‐AM pre‐treatment resulting in ∼10% greater cell death compared to DMSO controls. Statistical significance was assessed by one‐way ANOVA with Tukey's post hoc test (^****^ *P* < 0.0001).

In the context of our prior work showing mechanistically that chromatin domain‐mediated responsiveness is associated with chemoresistance and associated with worse prognosis in metastatic colon cancer, we hypothesized that intracellular ion chelation would be an effective mechanism to improve chemotherapeutic efficacy. Ion chelation dampens transcriptional responsiveness and, therefore, would limit the adaptive chemo‐evasive potential of cells. To test this hypothesis in HCT116 cells, we utilized both chelators and increased extracellular Mg^2+^ ions prior to the treatment with an IC50 dose of oxaliplatin for 48 h (Figure 7A). Our results were consistent with prior work demonstrating that chemotherapy selects for high D_n_ cells, resulting in higher D_n_ in surviving cell populations after 48 h (Figure 7B,C) [7, 9]. Pre‐treatment with extracellular MgCl_2_ increased the initial D_n_, leading to no additional response upon treatment with chemotherapy. However, pre‐treatment with divalent cation chelators not only decreases the initial D_n_, as reported in Figure 4A, but also amplifies the observed increase in D_n_ upon treatment with chemotherapy (Figure 7C,D). This finding was unexpected in the context of D_n_ returning to baseline after a few hours in cells that were not continuously exposed to chelation therapy. As such, it indicated that chelation‐mediated chromatin transformation impairs the endogenous domain‐mediated response system. Based on our previously published CPMC model, which predicts the transcriptional divergence of an ensemble of genes after a stimulus, this change in D_n_ would be expected to decrease cell viability upon treatment with chelators, which was proven here experimentally (Figure 7E) [81]. This behavior is consistent with prior in vitro studies demonstrating that chromatin compaction protects genomic DNA from chemical attacks [82]. In this context, ion‐mediated chromatin decompaction reduces this structural protection, thereby increasing susceptibility to oxaliplatin induced DNA damage. Remarkably, this indicates the crucial role of both chromatin domains and intranuclear divalent ion concentrations in cellular fitness. Although ion concentrations are not frequently measured in chemotherapeutic studies, this result demonstrates that we can use ionic modulation to predictably modulate chromatin conformations to alter the efficacy of standard therapeutic agents. As such, in addition to such technology having potential for adjuvant therapy, it suggests additional studies in patient tumor samples to study the role of ionic conditions in patient outcomes.

## 3 Discussion

This current study elucidates the intricate relationship between nuclear ions, particularly magnesium, and chromatin packing geometry in living human cancer cells. By utilizing the integrated nano‐ChIA imaging platform that pairs imaging of chromatin structure across length scales with cellular function, we demonstrated that selectively depleting or enriching intranuclear magnesium and calcium in living human cancer cells directly reshapes PD geometry across nanoscopic and mesoscopic length scales. ChromSTEM revealed that divalent cation depletion preferentially destabilizes nascent and weakly stabilized domains, reduces chromatin volume concentration, and diminishes heterochromatic cores (Figure 2). Conversely, magnesium enrichment enhances packing scaling, domain density, and structural maturation. Live‐cell PWS nanoscopy demonstrated that these changes in packing density occur within minutes, indicating that ionic regulation operates at timescales compatible with transcriptional control rather than long‐term epigenetic remodeling alone (Figure 5). Because divalent cations are essential cofactors for transcriptional machinery, ATP‐dependent remodeling enzymes, and chromatin‐associated biochemical processes, some degree of transcriptional and structural disruption is expected following large‐scale ionic perturbation. Our findings extend these established principles by demonstrating how ionic fluctuations reshape chromatin packing domain geometry across nanoscopic length scales in living cells. These observations suggest that nuclear ionic homeostasis may act as an important early‐stage biophysical regulator of the chromatin domain life cycle, tuning the rates of nascent domain formation, maturation, and decay to balance structural memory with transcriptional plasticity across the genome. In this framework, fluctuations in ionic strength could shift the threshold for domain stabilization, influencing how readily transcriptional programs are maintained or rewritten. Our modeling and live‐cell measurements suggest non‐linear responses to ionic perturbation, raising the possibility that chromatin operates near a stability threshold, such that even small ionic changes produce disproportionate domain packing consequences. Unlike epigenetic modifications, which require enzymatic turnover and may persist across cell divisions [83], ionic modulation operates on a rapid and reversible timescale [36, 84]. Nuclear ionic fluctuations may therefore provide a fast‐acting and reversable physiochemical layer of genome regulation that operates alongside slower epigenetic remodeling events.

Crucially, these geometric changes in PDs are functionally consequential. Transcriptomic profiling revealed widespread but patterned gene expression changes following cation modulation (Figure 6), consistent with the CPMC framework, linking D_n_ to transcriptional sensitivity. Gene expression sensitivity analysis suggests that altering domain geometry redistributes genes across transcriptionally optimal or suboptimal microenvironments, reshaping transcriptional output in a density‐dependent manner. In highly plastic systems such as cancer cells, the ability to form and stabilize new PDs appears essential for adaptive reprogramming under stress [9, 79]. Chelation‐mediated PD destabilization impaired this process and reduced chemoevasive capacity, linking ionic regulation of PD maturation directly to therapeutic vulnerability. Notably, ionic depletion does not simply “loosen” chromatin to increase accessibility. Rather, reduced ionic strength lowers the efficiency of nascent domain formation while accelerating structural decay of weakly stabilized PDs. Within this paradigm, the inability to create and mature new domains limits the formation of transcriptionally optimal microenvironments, thereby constraining adaptive transcriptional plasticity. This distinction helps reconcile why cation depletion reduces cellular resilience despite partial chromatin decompaction.

These mechanistic findings may have broad implications for understanding how chromatin geometry regulates cellular identity and adaptation across biological contexts. In aging, for example, progressive declines in ion homeostasis, mitochondrial function, or nuclear transport could impair the formation and stabilization of packing domains, contributing to erosion of transcriptional memory independent of DNA mutation burden [85, 86]. Similarly, cancer cells may operate near a critical threshold of domain instability, requiring continuous formation of nascent domains to sustain adaptive transcriptional programs [87, 88]; chelation may selectively disrupt this dynamic reassembly process, limiting the geometric flexibility necessary for chemoevasion.

The geometric sensitivity to ionic perturbation observed here is consistent with well‐established electrostatic principles governing chromatin structure. The high density of negative charges along the DNA backbone generates electrostatic repulsion, and divalent ions such as magnesium mitigate this through charge neutralization and ionic bridging [37, 81, 89]. By stabilizing DNA wrapping around histones and promoting inter‐nucleosomal interactions, magnesium enhances nucleosome integrity and facilitates higher‐order chromatin folding [16]. Conversely, chelation disrupts these stabilizing forces, reducing packing efficiency and domain stability. These electrostatic mechanisms provide a biophysical foundation for the domain‐level geometric shifts observed in this study. In addition to structural stabilization, magnesium supports enzymatic processes central to genome function. Mg‐ATP complexes serve as substrates for ATP‐dependent enzymes, including kinases, polymerases, and chromatin remodelers, and Mg^2^^+^ functions as a catalytic cofactor for transcriptional and chromatin‐modifying enzymes [90, 91]. Accordingly, the effects observed here likely reflect both direct electrostatic influences on chromatin geometry and the broader biochemical roles of divalent cations in nuclear function. Ionic modulation may therefore influence chromatin organization both directly, through electrostatic effects on nucleosome interactions, and indirectly, by shaping ATP‐dependent remodeling dynamics within densely packed regions. These electrostatic and enzymatic effects occur within a mechanically coupled nuclear environment [66]. By modulating osmotic gradients and ion flux, it can influence nuclear and cytosolic swelling, membrane tension, and mechanosensitive signaling pathways that reshape nuclear architecture. These coupled electrostatic and mechanical processes suggest that ionic regulation of chromatin operates within an integrated physical framework rather than an isolated biochemical variable.

Importantly, the observed changes in chromatin packing geometry were not mirrored by previously reported Hi‐C analyses examining the effects of ionic chelation on A/B compartments, TADs, and loop contacts, which were largely resilient to changes in salt concentrations [64]. If ionic modulation primarily alters geometric packing rather than genomic contact topology, this distinction may explain discrepancies with prior contact‐mapping studies. Population‐averaged contact frequency measurements may not fully capture the geometric packing and local crowding properties of chromatin in situ. While Hi‐C reports on proximity‐based interactions across a cell population, ChromSTEM resolves the physical density and hierarchical organization of chromatin within individual nuclei. Together, this study indicates that ionic modulation reshapes chromatin geometry without necessarily altering large‐scale contact maps, highlighting a distinction between chromatin packing domains and contact frequency–based organization.

Our findings suggest that modulation of nuclear ion concentrations offers a useful avenue for chromatin engineering and therapeutic innovation. Potential strategies include using ion‐regulating drugs like chelators and dopants to modulate nuclear ion concentrations. Electrical stimulation or low‐frequency electromagnetic radiation may also influence ionic distributions and chromatin dynamics without direct chemical intervention [92]. Cutting‐edge technologies, such as biodegradable hydropatches, hold particular promise for localized ionic manipulation, enabling precise therapeutic targeting of chromatin‐related diseases, promoting stem cell differentiation, and accelerating tissue repair with greater specificity and efficacy [93].

While this study demonstrates the utility of ionic manipulation for chromatin engineering in cancer cells, further exploration is needed to expand its applicability. Magnesium's integral role in maintaining cellular homeostasis highlights the need for careful consideration when manipulating its levels experimentally or therapeutically. Altering magnesium concentrations can have unintended consequences, such as disrupting energy metabolism, impairing DNA replication, or triggering inappropriate cellular signaling. Excessive magnesium depletion could hinder ATP production, leading to reduced energy availability for essential processes, while over‐supplementation might cause hypermagnesemia, affecting cardiovascular and neuromuscular functions [94]. Therefore, any intervention aiming to modify magnesium levels must be meticulously calibrated to avoid adverse effects. In research settings, this means using precise concentrations and exposure times to isolate specific outcomes without confounding variables. In clinical contexts, a balanced approach is necessary, considering the patient's overall ion status and monitoring for potential side effects. By acknowledging magnesium's multifaceted roles, scientists and clinicians can better design strategies that harness its benefits while mitigating risks, ultimately contributing to advances in biomedical research and therapeutic interventions. While magnesium is a central divalent cation in chromatin organization, other multivalent cations such as spermine and spermidine similarly compact DNA through electrostatic interactions [95, 96]. Because polyamines do not serve as essential cofactors for ATP‐dependent metabolism, they become potential targets for chromatin manipulation with distinct systemic consequences. Future research should integrate more complex models, such as 3D tissue cultures, organoids, or patient‐derived xenografts, to better simulate the tumor microenvironment and systemic interactions. In vivo models, including genetically engineered mice, could provide additional insights into the broader implications of chromatin‐targeted therapies on tumor progression, immune interactions, and therapeutic responses. Additionally, exploring other cancer types or diseases involving chromatin dysfunction will establish the generalizability of these approaches.

In conclusion, this study provides nanoscale insight into how magnesium and related ionic perturbations influence chromatin packing geometry and associated genome regulatory behavior, offering foundational insights for the development of chromatin‐targeted therapies. By leveraging these findings and integrating innovative methodologies, we can advance our understanding of chromatin dynamics and unlock new therapeutic possibilities for chromatin‐related diseases. Additionally, exploring the role of other multivalent cations like spermine and spermidine provides alternative avenues for chromatin manipulation that may avoid some of the broader metabolic consequences associated with magnesium. These insights collectively contribute to the growing field of chromatin engineering, with significant implications for cancer therapy and beyond.

## 4 Materials and Methods

### 4.1 Cell Culture

Human colorectal cancer cells (HCT116, ATCC, Cat# CCL‐247, RRID: CVCL_0291) were purchased from the American Type Culture Collection (ATCC) and cultured in McCoy's 5a modified medium (ThermoFisher Scientific, #16600082) supplemented with 10% fetal bovine serum (ThermoFisher Scientific, #16000044) and penicillin‐streptomycin (100 ug/ml; ThermoFisher Scientific, #15140122). Cells were maintained and imaged under standard culture conditions (5% CO_2_ and 37C) for the duration of the experiment. Experiments were performed on cells from passage 5 to 15.

### 4.2 Ionic Modulation

To modulate nuclear ion concentrations, HCT116 cells were treated with either 10 uM of the AM (acetoxy methyl) ester of the Ca^2+^ chelator BAPTA (1,2‐Bis(o‐aminophenoxy)ethane‐N,N,N’,N’‐teraacetic acid) [58, 97] (ThermoFisher, #B1205) or 20 uM the AM ester of APDAP (Aminophenoldiacetatephosphinate), a ligand with higher selectivity for Mg^2+^ [59, 98, 99]. Control cells were treated with equivalent volumes of vehicle (DMSO). The AM ester forms allowed diffusion across the cell membrane, with intracellular esterases releasing the active chelator. Chelation proceeded under standard conditions to maintain viability and minimize off‐target effects. Concentrations and incubation times were optimized from prior work to ensure effective chelation without undue toxicity. Additionally, to increase the concentration of nuclear Mg^2+^ ions, cells were treated with 1–10 mM MgCl_2_ solution (Sigma–Aldrich, #M1028) added extracellularly to cell culture media.

### 4.3 Ion Staining and Imaging

To assess the intracellular ion concentrations in HCT116 cells treated with cationic modulators, cells were labeled with ion‐specific fluorescent dyes for sodium, calcium, and magnesium. After ionic modulation, cells were washed twice with warm phosphate‐buffered saline (PBS) to remove residual media. To stain intracellular Mg^2+^, cells were incubated with 5 µM Magnesium Green AM (ThermoFisher, #M3735) diluted in PBS supplemented with 0.1% Pluronic F‐127 for 30 min at standard culture conditions. For Ca^2+^ labeling, cells were incubated with 5 µM Cal‐520 AM (AAT Bioquest, #21130) under the same conditions, and for Na^+^ labeling, cells were incubated with 5 uM CoroNa Green AM (ThermoFisher, #C36676). After staining, cells were washed three times with PBS to remove unbound dye and then fixed in 4% paraformaldehyde (PFA) (Electron Microscopy Sciences, #15710) in PBS for 10 min at room temperature. Cells were then counterstained with nuclear stain, DAPI (4’,6‐diamidino‐2‐phenylindole) (ThermoFisher, #D1306), and plasma membrane stain, CellMask (ThermoFisher, #C10046). Stained cells were protected from light and stored in PBS at 4°C. Stained cells were imaged using the Nikon SoRa Spinning Disk confocal microscope equipped with Hamamatsu ORCA‐Fusion Digital CMOS camera. Images were collected using a x60/1.42 NA oil immersion objective mounted to a x2.8 magnifier. After imaging, fluorescence intensity was quantified using ImageJ (NIH), with regions of interest (ROIs) defined to measure the nuclear fluorescence. Fluorescence intensity measurements were normalized to untreated controls to assess the impact of ionic modulators on intracellular Mg^2+^, Ca^2+^, and Na^+^ ion concentrations. Importantly, fluorescence intensities remained within the linear detection range and did not exceed saturation thresholds, ensuring that localized high‐intensity regions do not bias the integrated measurements. Three biological replicates were performed for each condition.

### 4.4 Spectral Partial Wave Spectroscopy (PWS) Imaging

Live‐cell PWS measurements were conducted under physiological conditions (37°C, 5% CO_2_) using a stage‐top incubator (In vivo Scientific, Stage Top Systems). Cells were imaged on a commercial inverted microscope (Leica DMIRB) equipped with a Hamamatsu Image‐EM CCD camera (C9100‐13) and a liquid crystal tunable filter (LCTF, CRi VariSpec) to acquire spectrally resolved images from 500 to 700 nm at 2‐nm intervals, as previously described [65, 67, 100]. Illumination was provided by a broadband white light LED source (Xcite‐120 LED, Excelitas), and the system was fitted with a long‐pass filter (Semrock BLP01‐405R‐25) and a 63× oil immersion objective (Leica HCX PL APO). Prior to imaging, cells were seeded on glass‐bottom dishes and allowed to re‐adhere for 24 h.

PWS imaging measures spectral interference patterns resulting from light scattering within the sample, which are sensitive to nanoscale variations in the local refractive index. Since the refractive index is proportional to macromolecular density, and chromatin density in particular, PWS provides a non‐invasive optical readout of 3D chromatin structure. According to the Gladstone‐Dale equation, the refractive index is a linear function of local molecular crowding. Therefore, the spectral variance (Σ) depends on the spatial density‐density autocorrelation function (ACF) of the medium's macromolecular mass density. Optical modeling and chromatin density autocorrelation functions used in this calculation are based on prior chromatin tomography work [7, 68, 100]. The spectral variance Σ is related to the chromatin fractal scaling through the power‐law behavior of the density ACF, from which the local packing scaling exponent D_a_(x,y) was computed at each pixel within the segmented nuclear region. The average nuclear chromatin packing scaling, D_n_, was then obtained by averaging D_a_ values across all pixels within the nuclear region. This value reflects the organization of chromatin into spatially segregated packing domains. For each experimental condition, nuclear D_n_ values were calculated for individual cells across biological replicates, and group‐level statistics were used to quantify changes in chromatin packing associated with treatment.

### 4.5 Dynamic PWS Measurements

Dynamic PWS measurements were performed as previously described [100, 101]. In this mode, wide‐field backscattered light at a fixed wavelength (550 nm) was recorded over time to generate a temporal image cube with dimensions (x, y, t). At each pixel, intensity fluctuations arise from the dynamic movement of macromolecular structures such as chromatin. Fractional moving mass (FMM) was calculated by measuring the temporal variance of intensity $\left(\sum_{t}^{2}\right)$ and normalizing it using a biophysical scattering model. This normalization converts raw intensity fluctuations into a quantitative estimate of nanoscale mass motion within the nucleus. As described in Gladstein et al. (2019), FMM is defined as:

(1) $$\sum_{t}^{2}\left(\frac{\pi \rho_{0}}{2\Gamma^{2}k^{3}n_{i}}\right){\left(\frac{NA_{i}}{NA_{c}}\right)}^{2}\;{\left(\frac{n_{1}}{n_{m}-n_{1}}\right)}^{2}=\rho_{0}\;V_{cm}\varphi =m_{c}\;\varphi =m_{f}\;$$

With this normalization, $\sum_{t}^{2}$ is equivalent to *m_f_*, which measures the mass moving within the sample. This value is calculated from the product of the mass of the typical moving cluster (*m_c_*) and the volume fraction of mobile mass (ϕ). *m_c_* is obtained by *m_c_* = *V_cm_* ρ_0_, where *V_cm_* is the volume of the typical moving macromolecular cluster. To calculate this normalization, we approximate n_m_ = 1.43 as the refractive index (RI) of a nucleosome, *n*_1_ = 1.37 as the RI of a nucleus, *n_i_* = 1.518 as the refractive index of the immersion oil, and ρ_0_ = 0.55 g cm^−3^ as the dry density of a nucleosome. Additionally, *k*  = 1.57 × 105 cm^−1^ is the scalar wavenumber of the illumination light, and Γ is a Fresnel intensity coefficient for normal incidence. *N* *A_c_* = 1.49 is the numerical aperture (NA) of collection and *N* *A_i_* = 0.52 is the NA of illumination. As stated previously [100], $\sum_{t}^{2}$ is sensitive to instrument parameters such as the depth of field and substrate refractive index. Instrument‐specific parameters such as depth of field and substrate RI were corrected using previously determined calibration constants5858. Pixelwise FMM values were calculated for each nucleus, and distributions were compared across conditions to quantify treatment‐induced changes in chromatin mobility.

### 4.6 STORM Sample Preparation and Imaging

HCT116 cells were seeded on 35‐mm glass‐bottom dishes and cultured to ∼70% confluence. Cells were fixed in 3% paraformaldehyde and 0.1% glutaraldehyde in phosphate‐buffered saline (PBS) for 10 min at room temperature, followed by quenching in 0.1% sodium borohydride for 7 min. After two PBS washes (5 min each), cells were permeabilized and blocked in PBS containing 0.2% Triton X‐100 and 3% bovine serum albumin (BSA) for 20 min. To label constitutive heterochromatin, cells were incubated with for 2 h with anti‐H3K9me3 antibody (Abcam, cat# ab176916, RRID: AB_2797591) at 2.5 µg/mL in blocking buffer. To label transcriptionally active chromatin regions, cells were incubated for 2 h with anti‐RNA polymerase II phospho‐S2 antibody (Abcam, cat# ab252854, RRID: AB_2631402) at 2.5 µg/mL in blocking buffer. After three washes in PBS with 0.1% Triton X‐100 and 0.2% BSA, cells were incubated with Alexa Fluor 647‐conjugated secondary antibody (H3K9me3) (Thermo Fisher, cat# A‐21245, RRID: AB_2535813) or Alexa Fluor 488‐conjugaed antibody (POLR2A) (Thermo Fisher, cat# A‐11006, RRID: AB_2534074) at 2.5 µg/mL for 40 min. Cells were washed twice in PBS and prepared for imaging in STORM buffer containing 0.5 mg/mL glucose oxidase (Sigma‐Aldrich), 40 µg/mL catalase (Roche), and 100 mg/mL glucose in TN buffer (50 mM Tris, pH 8.0, and 10 mM NaCl). Super‐resolution imaging was performed using a Nikon Eclipse Ti‐U inverted microscope equipped with a 100×/1.49 NA oil immersion objective (SR APO TIRF, Nikon) and an iXon Ultra 888 electron‐multiplying CCD camera (Andor). A 637‐nm laser (Obis, Coherent) provided excitation at the sample plane with an average power of 3–10 kW/cm^2^. At least 8000 frames were acquired per field of view with a 20 ms exposure time.

### 4.7 ChromEM Sample Preparation and 3D Tomographic Imaging

ChromEM sample preparation was adapted from published ChromEMT and ChromSTEM protocols using reagents listed in Table S1 [8, 19, 60]. Cells were washed three times with calcium‐ and magnesium‐free Hank's Balanced Salt Solution, followed by fixation in 2.5% glutaraldehyde and 2% paraformaldehyde for 30 min at room temperature and an additional 30 min at 4°C. Fixed cells were blocked for 15 min, stained with 10 mM DRAQ5 for 10 min, and rinsed three times with blocking buffer (5 min each). Samples were then photo‐bleached in diaminobenzidine (DAB) solution for 5 min and washed five times (2 min each) before staining with reduced osmium for 30 min. Following heavy metal staining, samples were sequentially dehydrated in ethanol and embedded in Durcupan ACM resin. Ultrathin sections (120 nm) were cut using a Leica UC7 ultramicrotome with a 35° Diatome diamond knife and mounted on 2 × 0.5 mm copper slot grids coated with Formvar‐carbon film. Gold nanoparticles were applied to both surfaces to serve as fiducial markers for tomographic alignment. Electron tomography was performed on a Hitachi HD2300 scanning transmission electron microscope (STEM) operated in high‐angle annular dark field (HAADF) mode at 50,000× magnification. Samples were pre‐exposed for 30 min prior to imaging. Dual‐axis tilt series were collected from −60° to +60° at 2° increments along two approximately orthogonal axes. Tilt series were aligned using IMOD and reconstructed using the TomoPy penalized maximum likelihood algorithm with weighted linear and quadratic penalties. The two reconstructed volumes were merged in IMOD. Gold nanoparticles were digitally removed prior to final reconstruction using the same pipeline. To enhance visualization, the top and bottom 0.1% of voxel intensity values were removed, and the remaining intensities were normalized to a scale of 0 to 1.

### 4.8 ChromSTEM Domain Analysis

Chromatin domains were identified and analyzed using previously described methods [8, 19]. Mass scaling behavior was assessed by selecting random seed points within the chromatin volume and calculating the integrated chromatin density within expanding radial windows. The relationship between chromatin mass and radius was plotted on a log–log scale to extract the local mass scaling exponent, *D*. The first derivative of this plot was used to identify transitions between distinct scaling regimes; regions beyond 300 nm were classified as part of the supra‐domain regime and excluded from domain‐level analysis. Domain centers were defined as local maxima in chromatin density after applying Gaussian filtering and CLAHE (Contrast‐Limited Adaptive Histogram Equalization) for local contrast enhancement. For each identified center, a square region with a half‐width of 300 nm was extracted for localized domain analysis. Within this window, an 11 × 11‐pixel grid was used to further refine center selection and improve sampling across the nucleus. The linear region of the log–log mass‐radius curve was identified using MATLAB's *ischange* function. Domain size (*R_f_*) was defined as the radius at which any of the following criteria were met: (i) the mass‐radius curve deviated by more than 5% from its linear fit, (ii) the local mass scaling exponent reached 3, or (iii) chromatin density reached a local minimum. Chromatin volume concentration (CVC) was calculated as the fraction of chromatin‐occupied volume within the domain. This was determined using Otsu's binarization algorithm applied to grayscale density maps after CLAHE preprocessing in ImageJ. Packing efficiency, A, is calculated as described previously:

(2) $$I=A{\left(\frac{R_{f}}{R_{\min}}\right)}^{D}$$

where *I* is the average chromatin intensity of a domain, *R_f_* is the domain size, R_min_ is the estimated smallest unit of random chromatin polymer chain (10 nm), and *D* is the packing scaling exponent of the domain packing behavior.

### 4.9 RNAseq

Total RNA was extracted from HCT116 cells treated under three conditions: DMSO control, 1‐h APDAP‐AM chelation, and 1‐h BAPTA‐AM chelation. Each condition was performed with three biological replicates. Stranded mRNA sequencing and library preparation were carried out by the Northwestern University NUSeq Core Facility. Libraries were sequenced using the Illumina HiSeq 4000 platform to generate single‐end 50 bp reads. Raw reads in FASTQ format were quality‐checked using FastQC, and a comprehensive multi‐sample summary was produced using MultiQC. Reads were aligned to the human genome (hg38) using STAR, and transcript quantification was performed using RSEM to estimate TPM (transcripts per million) values. Gene‐level count data were imported using the tximport package in R. Genes with zero abundance and zero length across all samples were removed prior to analysis. Differential gene expression analysis was carried out using DESeq27777, with a design formula modeling treatment condition. Log2 fold changes were estimated using shrinkage estimators, and statistical significance as asses with Wald test. Multiple hypothesis testing was corrected using the Benjamini–Hochberg method. Genes with an adjusted p‐value < 0.05 and a base mean >50 were considered significantly differentially expressed. Upregulated and downregulated genes were defined as having log_2_ fold changes >1 and <−1, respectively. Ensembl gene identifiers were mapped to gene symbols using the org.Hs.eg.db annotation package.Variance‐stabilized expression values were used to visualize top differentially expressed genes via heatmaps, and principal component analysis (PCA) was conducted to evaluate sample clustering by condition. Gene Ontology (GO) enrichment analysis was performed using the clusterProfiler package with the enrichGO function. Enrichment was assessed against the expressed gene set background with an FDR cutoff of 0.05. GO categories were ranked based on adjusted p‐value and gene count.

### 4.10 Sensitivity Curves

Gene expression sensitivity to changes in chromatin structure was modeled using the Chromatin Packing Macromolecular Crowding (CPMC) framework [7]. According to this model, the average gene expression level (*E*) for a group of genes at a given chromatin packing scaling value (D_n_) can be expressed as the product of two components:

(3) $$E=P_{s}\;\cdot \bar{\varepsilon }$$

where P_s_ is the probability that a gene lies on the accessible surface of a packing domain, and $\bar{\varepsilon }$ is the average mRNA expression rate for those accessible genes. The value of *P*_s_ is determined by the fractal geometry of chromatin domains, specifically the power‐law mass scaling behavior. For a packing domain of genomic size *N_f_* and scaling exponent *D*, *P*_s_ is given by:

(4) $$P_{s}={\left(N_{f}\right)}^{-1/D}$$

This reflects the accessible surface of chromatin. The average transcription rate $\bar{\varepsilon }$ is derived from a systems biology model of gene expression under macromolecular crowding. Transcription is modeled as a network of chemical reactions influenced by both transcriptional regulators and local crowding conditions. The average mRNA production rate is obtained by integrating over the crowding density distribution ϕ:

(5) $$\bar{\varepsilon }=\int \varepsilon \left(\overset{⇀}{m},\phi \right)f\left(\phi \right)d\phi$$

where ε($\overset{⇀}{m}$,ϕ) is the local expression rate determined by molecular regulators $\overset{⇀}{m}$, and *f*(ϕ) is the probability density function of crowding. Assuming Gaussian‐distributed crowding density, *f*(ϕ) is approximated as:

(6) $$f\;\left(\phi \right)=\frac{1}{\sqrt{2\pi {\sigma_{\phi ,in}}^{2}}}e^{\frac{{\left(\phi -\phi_{in,0}\right)}^{2}}{2{\sigma_{\phi ,in}}^{2}}}$$

where $\phi_{in,0}$ is the mean crowding density within a transcriptional interaction volume, $\sigma_{\phi ,in}^{2}$ is the variance in crowding density. The crowding variance is determined by chromatin packing scaling D_n_:

(7) $${\sigma_{\phi }}^{2}=\phi_{in,0}\;\left(1-\phi_{in,0}\right){\left(\frac{r_{min}}{L_{in}}\right)}^{3-D_{n}}$$

where r_min_ is the size of an elementary chromatin particle (e.g., a nucleotide), L_in_ is the length scale of the transcriptional interaction volume. This framework links chromatin physical properties (packing density and spatial organization) to the probabilistic regulation of gene expression, allowing prediction of transcriptional changes as a function of structural chromatin remodeling.

To experimentally evaluate the sensitivity predicted by the CPMC model, chromatin packing scaling (*D_n_*) was obtained from live cell Partial Wave Spectroscopic (PWS) microscopy, and gene expression levels (E) were quantified from RNA‐Seq data (TPM‐normalized). Differentially expressed genes (adjusted p‐value < 0.01) were grouped into quantiles based on baseline expression. For each quantile, the average change in log‐transformed expression between treatment and control conditions (Δln (*E*)) was divided by the normalized change in chromatin packing ($\Delta \ln(D_{n}$)) to yield an empirical sensitivity estimate:

(8) $$Se=\frac{\Delta \ln\left(E\right)}{\Delta \ln\left(D_{n}\right)}\approx \frac{\Delta \ln\left(E\right)}{\Delta \ln\left(D_{n}/D_{n,0}\right)}$$

These values were plotted against the normalized log expression $\ln(E_{control}/\bar{E_{control}})$ to generate sensitivity curves, representing the degree to which transcriptional output responds to structural perturbations in chromatin packing. This empirical implementation directly tests the bidirectional sensitivity behavior predicted by the CPMC model, where increases in chromatin packing scaling amplify transcriptional divergence, enhancing gene expression of highly active genes while suppressing transcription of lowly expressed genes.

### 4.11 Viability Assay

Cell viability was assessed using the PrestoBlue assay (Thermo Fisher Scientific, #A13262). HCT116 cells were seeded into black‐walled, clear‐bottom 96‐well plates and allowed to adhere overnight. Cells were pretreated with either APDAP‐AM or BAPTA‐AM for 60 min, followed by treatment with 7.5 µM oxaliplatin (Sigma‐Aldrich, #O9512) for 24 h under standard culture conditions. Following treatment, 10 µL of PrestoBlue reagent was added directly to each well containing 90 µL of culture medium. Plates were incubated for 30 min at 37 °C, and absorbance was measured at 570 nm with a 600 nm reference using a Multiskan SkyHigh microplate reader (Thermo Fisher Scientific). Wells containing medium and reagent without cells were used as blank controls to correct for background signal. Cell viability was calculated as:

(9) $$Cell\;Viability\;\left(\%\right)=\left(\frac{Abs_{treated}}{Abs_{control}}\right)\;\times 100$$

where Abs_treated_ is the background‐corrected absorbance of treated wells and Abs_control_ is that of DMSO‐treated control wells.

### 4.12 Phenomenological Chromatin Packing Domain Model

To study the physical regulation of chromatin organization under ionic stress, we extended and used a previously developed phenomenological model of loop domain formation that captures the physical interplay between chromatin crowding, transcriptional activity, and polymer folding dynamics. This model is grounded in three core principles. Namely (1) Loop returns (originating from transcription or cohesion‐mediated loop extrusion) generate localized chromatin condensates, (2) macromolecular crowding influences the ability of enzymes and regulatory proteins to access and modify chromatin, and (3) Transcriptional efficiency, which exhibits a non‐monotonic dependence on local chromatin density, arises from the trade‐off between diffusivity and reaction free energy.

Within this model, chromatin is treated as a flexible polymer confined within a nuclear subvolume that is heterogeneously packing within the nucleus and forms PDs. PDs are assumed to follow a fractal polymer scaling law, where the local chromatin volume fraction (Equation 1) decays radially as a function of the center of the PD

(10) $$\phi \;\left(r\right)=p_{0}\;{\left(r/r_{min}\right)}^{D-3}$$

where *r_min_* =  10 *nm* is the minimal chromatin chain element length, *p*_0_ is the chromatin volume concentration at the core of a PD (*r*  =  0), and *D* is the domain's packing scaling exponent which governs how chromatin mass scales with domain size. This formulation enables quantification of both crowding and spatial organization within PDs.

The model consists of a system of coupled ordinary differential equations (ODEs) that describe the temporal evolution of chromatin states.

(11) $$N=N_{t}+N_{\Delta s}$$

(12) $$\frac{dN_{t}\left(t\right)}{dt}=\phi_{e}\;\left(t\right)\Gamma_{POL2}-N_{t}\left(t\right)\Gamma_{TOP}$$

(13) $$\frac{dN_{\Delta s}}{dt}=\Gamma_{\Delta s}\;*\left[N_{max}-N\left(t\right)\right]$$

(14) $$\phi \;\left(t\right)=\phi_{e}\;\left(t\right)+\phi_{h}\;\left(t\right)=\frac{3}{D\left(t\right)}\;\times A\left(t\right){\left(\frac{r_{min}}{r}\right)}^{3-D\left(t\right)}$$

(15) $$\frac{d\phi_{h}\left(t\right)}{dt}-\;\frac{d\phi_{e}\left(t\right)}{dt}=\phi_{e}\;\left(t\right)\Gamma_{h}-\phi_{h}\left(t\right)\Gamma_{e}$$

(16) $$\Gamma_{\Delta s}=B_{\Delta s}\;*e^{\left(\frac{-\phi_{h}}{\phi *ps}\right)}$$

Here, *N_t_* (*t*) is the fraction of chromatin engaged in transcriptional loop returns, *N*_Δ*s*_(*t*) corresponds to the fraction of chromatin undergoing entropic (non‐enzymatic) returns, $\phi_{h}\left(t\right)$ and $\phi_{e}\left(t\right)$ describe the chromatin volume fractions of heterochromatin and euchromatin, and *D*(*t*) is the time‐varying packing scaling exponent describing how the polymer scales with size. Returns from either through transcriptional mechanisms or entropic crosslinking, each associated with distinct biophysical feedback. Equation 3 governs the time evolution of transcriptional returns, controlled by two rate constants: Γ_*POL*2_, representing RNA Polymerase II activity, and Γ_*TOP*_, representing Topoisomerase‐mediated loop relaxation. These rate constants exhibit non‐monotonic dependencies on local chromatin composition, particularly the balance between euchromatin and heterochromatin volume fractions. Enzymatic activity is modeled as a function of local crowding, when the return formation reflects the trade‐off between chromatin accessibility and chromatin density. Accessibility is further modulated by chromatin folding via a mechanistic function *f*(*D*(*t*),  *p_c_*, *p*_0_, *r_min_*, *r*) where *p_c_* defines the enzyme‐specific crowding tolerance. A complete breakdown of the model and equations, including explicit equations for the rate constant and folding function, can be found in *Almassalha* et al. [16].

The full set of ODE's was implemented and numerically solved using Wolfram Mathematica 13.2 over a 12‐h time segment. The following initial conditions were set to $D\;\left(0\right)=2.2,\;\phi_{h}\;\left(0\right)=0.05,\;N_{t}\;\left(0\right)=0$ and *N_s_* (0) = *N_max_* , where *N_max_* represents the experimentally measured maximum return capacity from ChromSTEM‐derived PDs. To model the evolution of PDs under distinct ionic environments, we systematically modulated key parameters reflecting known physical responses of chromatin to divalent cations, such as Mg^2+^. More specifically, we increased the initial packing efficiency of the starting euchromatic chain, $\phi_{0,e}$, under high ionic conditions to account for enhanced charge screening, increased the rate constant Γ_*TOP*_ due to Mg^2+^’s role as a catalytic cofactor and its experimentally observed linear relationship with DNA relaxation dynamics, decreased the crosslinking memory threshold (*ps*) to reflect stronger entropic stabilization at higher ion concentrations, and slightly elevated the maximum loop capacity (*N_max_*) in accordance with experimentally observed increases in packing scaling (ChromSTEM). A complete list of parameters used for each ionic condition is detailed in Table S2.

### 4.13 Statistics

All quantitative data are presented as mean ± standard deviation (SD), and scatter plots were used to visualize data distributions where appropriate. Statistical analyses were performed using GraphPad Prism (GraphPad Software, San Diego, CA). For comparisons between two groups (e.g., DMSO vs. BAPTA), statistical significance was assessed using a two‐sided Student's *t*‐test. For comparisons involving more than two groups, a one‐way analysis of variance (ANOVA) was performed followed by Tukey's post‐hoc test to identify pairwise differences. For chromatin packing scaling (D_n_), multiple group comparisons were evaluated using ANOVA with Bonferroni correction to account for multiple hypothesis testing. In all analyses, p < 0.05 was considered statistically significant. For RNA‐seq data, differential gene expression was evaluated using DESeq2, and significance was determined using false discovery rate (FDR)‐adjusted p‐values, calculated with the Benjamini–Hochberg procedure. Genes with adjusted p < 0.05 were considered significantly differentially expressed. All experiments were independently repeated at least twice with biological replicates to ensure reproducibility of results.

## Author Contributions

Conceptualization was carried out by C.L.D., R.J.N., I.S., and V.B. The original draft of the manuscript was written by C.L.D. and P.C.G. Review and editing of the manuscript were performed by C.L.D., P.C.G., L.M.A., W.S.L., N.A., I.C.Y., J.F., C.H., G.W., R.J.N., I.S., and V.B. Methodology was developed by C.L.D., P.C.G., L.M.A., W.S.L., R.G., N.A., G.W., I.S., and V.B. Resources were provided by L.M.A., G.W., I.S., and V.B. Funding acquisition was managed by L.M.A., I.S., and V.B. Supervision was provided by I.S. and V.B. Project administration was conducted by I.S. and V.B. Investigation was performed by C.L.D., P.C.G., W.S.L., R.G., N.A., C.H., and S.J. Validation was carried out by C.L.D. and P.C.G. Visualization was created by C.L.D. and P.C.G. Data curation was performed by C.L.D. and P.C.G.

## Conflicts of Interest

The authors declare no conflicts of interest.

## Supporting information




**Supporting File**: advs76786‐sup‐0001‐SuppMat.docx.

## Data Availability

RNA sequencing data that support the findings of this study are openly available in Dryad at https://datadryad.org/, reference number https://doi.org/10.5061/dryad.0k6djhbg5. Custom scripts for data analysis and visualization are available at: https://github.com/BackmanLab. All other data are included in the article and/or supporting information.
