# Supplementary material for: Chromatin Packing Domain Engineering Through the Manipulation of Nuclear Cationic States
Source: Adv Sci (Weinh). 2026 Aug 13;13(58):e76786. doi: 10.1002/advs.76786 (PMC13470300; doi:10.1002/advs.76786)
Supplement: Supplementary file 1 [file ADVS-13-e76786-s001.docx]

**SUPPLEMENTARY DATA**

Table S1. Reagents in ChromEM Staining

| Reagent | Formula |
| --- | --- |
| Washing solution | Hank’s balanced salt solution without calcium and magnesium |
| Fixation solution | 2.5% EM grade glutaraldehyde  2% paraformaldehyde  2mM calcium chloride  0.1M sodium cacodylate buffer, pH=7.4 |
| Blocking solution | 10mM glycine  10mM potassium ferrocyanide  0.1M sodium cacodylate buffer, pH=7.4 |
| DNA staining solution | 10mM DRAQ5  0.1% saponin  0.1M sodium cacodylate buffer, pH=7.4 |
| Photo-staining solution | 2.5mM 3,3’-diaminobenzidine tetrahydrochloride (DAB)  0.1M sodium cacodylate buffer, pH=7.4 |
| Reduced osmium staining solution | 2% osmium tetroxide  1.5% potassium ferrocyanide  2mM calcium chloride  0.15M sodium cacodylate buffer, pH=7.4 |

Table S2. List of Parameters in Phenomenological Chromatin Packing Domain Model

| **Parameter** | **Description** | **Low Divalent Ion Concentrations** | **High Divalent Ion Concentrations** |
| --- | --- | --- | --- |
| $A_{POL2}$ | Scaling factor for the max rate of RNA Polymerase II | 2 | 2 |
| $A_{TOP}$ | Scaling factor for the max rate of Topoisomerase | 0.25 | 0.47 |
| $A_{h}$ | Scaling factor for the max rate of heterochromatic modifiers | 1 | 1 |
| $A_{e}$ | Scaling factor for the max rate of euchromatic modifiers | 1 | 1 |
| $A_{\Delta s}$ | Scaling factor for the max rate of crosslinking proteins | 1 | 1 |
| $\sigma_{POL2}$ | Chromatin volume fraction limit in which RNA Polymerase II can no longer diffuse | 0.5 | 0.5 |
| $\sigma_{TOP}$ | Chromatin volume fraction limit in which Topoisomerase can no longer diffuse | 0.7 | 0.7 |
| $\sigma_{h}$ | Chromatin volume fraction limit in which heterochromatic modifiers can no longer diffuse | 1 | 1 |
| $\sigma_{e}$ | Chromatin volume fraction limit in which euchromatic modifiers can no longer diffuse | 0.5 | 0.5 |
| $ps$ | Crosslinking strength of the PD core | 0.10 | 0.15 |
| $r_{min}$ | The diameter of the smallest element in a chromatin chain: a nucleosome | 10 nm | 10 nm |
| $r$ | The average radius of a PD (experimentally attained by ChromSTEM) | 105.7 nm | 105.7 nm |
| $X/X_{0}$ | Ratio of genomic distance | 24 | 24 |
| $N_{0}$ | The loop volume fraction for the smallest element of the chromatin chain | 1 | 1 |
| $N_{max}$ | The optimal loop capacity within the volume | 0.026 | 0.029 |
| $\phi_{0,e}$ | Proportion of the chromatin chain that is euchromatic | 0.4 | 0.55 |

**SI Figure 1: Quantification of intracellular magnesium using 3D image reconstruction and CTCF analysis.** (A) Representative z-stack slices from immunofluorescence images showing green (magnesium), blue (DAPI for nucleus), and red (CellMask for cell membrane) fluorescence channels. (B) 3D automated segmentation of the nucleus (blue) and cell body (red) across the xy-, xz-, and yz-planes used to define regions of interest for signal quantification. (C) Formula used to calculate the corrected total cell fluorescence (CTCF), along with an example of z-stack data processed using this method to quantify intracellular magnesium levels.

**SI Figure 2: Manipulation of nuclear Ca^2+^ and Na^+^ ions via chelation and ion loading.** (A) Representative images of HCT116 cells treated with ion chelators (BAPTA-AM, APDAP-AM) or exogenous Mg^2+^ (MgCl_2_) and stained for intracellular Ca^2+^ ion indicator (Cal-520 AM) and DNA (DAPI). (B) Representative images of HCT116 cells treated with ion chelators or exogenous Mg^2+^ and stained for intracellular Na^+^ ion indicator (CoroNa Green AM) and DNA (DAPI). Scale bars are 10 µm in all images.

**SI Figure 3: Divalent cation depletion alters H3K9me3 heterochromatin organization and RNA Polymerase II localization.** (A–B) Representative multiplexed STORM images demonstrating the spatial organization of H3K9me3-marked heterochromatin (magenta) and active RNA Polymerase II (blue) in multiple HCT116 nuclei under control conditions (A) and following treatment with APDAP-AM for 60 minutes (B). Images are shown to illustrate the consistency of chromatin and transcriptional labeling patterns across nuclei and provide a broader contextual view of the nanoscale features quantified in Figure 3. Scale bars are 4 µm in all images.

**SI Figure 4: Transcriptomic response to divalent cation chelation in HCT116 cells.** (A) Principal component analysis (PCA) of RNA-seq data showing distinct clustering of biological replicates for each treatment group (DMSO, BAPTA-AM, and APDAP-AM), indicating consistency within groups and separation between conditions. (B) Heatmap of the top upregulated and downregulated genes in chelation-treated cells relative to DMSO controls, with all three replicates per condition displayed. (C) Sensitivity curves showing that genes upregulated in control cells tend to become more highly expressed following chelation, while downregulated genes become further suppressed, consistent with chelation amplifying pre-existing transcriptional biases. (D) Gene ontology (GO) enrichment analysis of significantly upregulated and downregulated cellular processes in chelated cells compared to DMSO, highlighting changes in pathways related to transcription, biosynthesis, and metabolic regulation.
